# Integrated analysis of mRNA-seq and miRNA-seq in calyx abscission zone of Korla fragrant pear involved in calyx persistence

**DOI:** 10.1186/s12870-019-1792-0

**Published:** 2019-05-09

**Authors:** Li Ma, Li Zhou, Shaowen Quan, Hang Xu, Jieping Yang, Jianxin Niu

**Affiliations:** 1 Department of Horticulture, College of Agriculture, Shihezi University, Shihezi, 832003 Xinjiang China; 2 Xinjiang Production and Construction Corps Key Laboratory of Special Fruits and Vegetables Cultivation Physiology and Germplasm Resources Utilization, Shihezi, 832003 Xinjiang China

**Keywords:** Korla fragrant pear, Transcriptome, miRNA, Calyx abscission, Gene expression regulation

## Abstract

**Background:**

The objective of this study was to characterize molecular mechanism of calyx persistence in Korla fragrant pear by transcriptome and small RNA sequencing. Abscission zone tissues of flowers at three stages (the first, fifth and ninth days of the late bloom stage), with 50 mg/L GA_3_ (calyx persistence treatment, C_1, C_5, C_9) or 500 mg/L PP_333_ (calyx abscission treatment, T_1, T_5, T_9), were collected and simultaneously conducted transcriptome and small RNA sequencing.

**Results:**

Through association analysis of transcriptome and small RNA sequencing, mRNA-miRNA network was conducted. Compared calyx persistence groups with calyx abscission groups during the same stage, 145, 56 and 150 mRNA-miRNA pairs were obtained in C_1 vs T_1, C_5 vs T_5 and C_9 vs T_9, respectively; When C_1 compared with C_5 and C_9, 90 and 506 mRNA-miRNA pairs were screened respectively, and 255 mRNA-miRNA pairs were obtained from the comparison between C_5 and C_9; When T_1 compared with the T_5 and T_9, respectively, 206 and 796 mRNA-miRNA pairs were obtained, and 383 mRNA-miRNA pairs were obtained from the comparison between T_5 and T_9. These mRNAs in miRNA-mRNA pairs were significantly enriched into the terpenoid backbone biosynthesis, photosynthesis - antenna proteins, porphyrin and chlorophyll metabolism, carotenoid biosynthesis, zeatin biosynthesis and plant hormone signal transduction. In addition, we obtained some key genes from miRNA-mRNA pairs that may be associated with calyx abscission, including protein phosphatase 2C (psi-miR394a-*HAB1*), receptor-like protein kinase (psi-miR396a-5p-*HERK1*), cellulose synthase-like protein D3 (psi-miR827-*CSLD3*), beta-galactosidase (psi-miR858b-β-galactosidase), *SPL*-psi-miR156j/157d, abscisic acid 8′-hydroxylase 1 (psi-miR396a-5p-*CYP707A1*) and auxin response factor (psi-miR160a-3p-*ARF6*, psi-miR167d-*ARF18*, psi-miR167a-5p-*ARF25*), etc.

**Conclusion:**

By integrated analysis mRNA and miRNA, our study gives a better understanding of the important genes and regulation pathway related to calyx abscission in Korla fragrant pear. We have also established the network of miRNA-mRNA pairs to learn about precise regulation of miRNA on calyx abscission.

**Electronic supplementary material:**

The online version of this article (10.1186/s12870-019-1792-0) contains supplementary material, which is available to authorized users.

## Background

Korla fragrant pear (*Pyrus sinkiangensis* Yu), is an ancient regional high-quality variety in Xinjiang Uyghur Autonomous Region, China. In Korla fragrant pear, the calyx is deciduous in some flowers but persistent in others. A persistent calyx is the main cause of deformed fruit in Korla fragrant pear. This can negatively affect pear shape and quality and directly affect the economic return from Korla fragrant pear [1].

Researchers have studied the relationship between calyx persistence in Korla fragrant pear and tree morphology [2], plant growth regulators [3, 4], root stock type [5], pollination [6], pruning [7] and light [8]. Ma et al. observed the difference of calyx tube microstructure at calyx developing stage after spraying PP_333_ or GA_3_ at florescence in Korla fragrant pear. It was found that the average area of vascular bundle of calyx tube tissue was bigger and many sieve tube cells and idioblasts gradually appeared when the tree was treated with GA_3_, which provided calyx with nutrients and moisture, prevented calyx tube from abscission layer formation and resulted in the formation of persistent calyx fruit at last. When the tree was treated with PP_333_, calyx tubes of young fruit only had vessels in vascular bundle. Abscission layer appeared at the late young fruit of calyx tube developing stage, and finally the calyx tube broke off and young fruit became no calyx fruit at last [9]. Calyx persistence was closely related to GA_3_ content in pear calyx. High GA_3_ content in calyx was conducive to the calyx persistence, on the contrary, low GA_3_ content in calyx was beneficial to the calyx abscission [10–12]. Previously study showed that the ability of calyx dropping became stronger with its order place rising in the same inflorescence. The calyx persistence rate from the first to the forth position gradually decreased, and the calyx abscission rate gradually increased [2]. Calyx persistence also related with tree vigor. There were more calyx persistence fruits in pear trees with vigorous tree vigor, and less calyx persistence fruits in pear trees with weak tree vigor [13]. Besides, some studies also have analyzed the molecular mechanism of calyx persistence. It is suggested that the *MYB*-like gene (*kfpMYB*) is involved in calyx persistence [14–16]. Sun et al. identified three *SPL* genes associated with the sepal development by high-throughput sequencing [17]. Tian et al. isolated and cloned the pectate lyase *PsPL* gene in Korla fragrant pear [18]. Qi et al. identified the candidate genes in calyx abscission zone of Korla fragrant pear involved in the calyx abscission process by digital transcript abundance measurements [19]. Pei et al. identified more than fifty genes related to calyx persistence using RNA-seq and digital gene expression (DGE) techniques [20]. Zhou et al. identified several miRNAs involved in calyx persistence using small RNA sequencing [21]. Instead of focus on the abscission zone of Korla fragrant pear, most researches have studied the calyx and ovary. Moreover, these studies on the molecular mechanism of calyx abscission were performed through separated applications of transcriptomes and small RNA sequencing, yet the association analysis combining both omics has not been reported.

MicroRNAs (miRNAs) are short, noncoding RNAs, about 19–24 nucleotides in length, with variable sequence complementarity to longer target RNAs [22]. However, the current determination of miRNA targets remains a significant challenge because the target sequence of miRNAs may not be conserved [23]. And the interactions between the miRNAs and their target genes may be one-to-many or many-to-one, rather than strictly one-to-one, resulting in a very large number of potential regulatory effects [24]. In addition, estimating the regulatory function of miRNAs based on the function of target mRNA predicted by bioinformatics software is inaccurate [25]. There are a potentially more reliable method for predicting target genes of miRNA: firstly, small RNA sequencing is used to identify and predict the target genes of differentially expressed miRNAs, then RNA-seq technology is used to screen differentially expressed mRNAs, and finally overlapped genes between target genes and differentially expressed mRNAs were selected to determine the regulatory relationship of miRNAs and mRNAs [26].

In this study, we have focused on the calyx abscission zone of Korla fragrant pear and analyzed miRNA and mRNA expression profiles between calyx persistence group and calyx abscission group using mRNA-seq and miRNA-seq in order to elucidate the molecular mechanisms of calyx persistence. Besides, we combined differentially expressed mRNAs and differentially expressed miRNAs and determine the critical miRNA-mRNA networks and pathways in calyx abscission zone of Korla fragrant pear involved in calyx persistence. This is the first report on integrated analysis of mRNA-seq and miRNA-seq in Korla fragrant pear and as such offers deeper insight into the molecular mechanisms of calyx persistence.

## Methods

### Plant material and treatment

The plant materials used in this study were obtained in spring 2018 at the Shayidong Horticulture Field, Korla, Xinjiang Province. Two uniform twenty-year-old Korla fragrant pear trees were selected and treated with either 50 mg/L GA_3_ (calyx persistence treatment) or 500 mg/L PP_333_ (calyx abscission treatment) at full bloom stage. Flowers were collected on the first, fifth and ninth days of the late bloom stage, respectively. The first flower to open in clusters on trees has a persistent calyx. The fourth flower to open in clusters from trees has a deciduous calyx. Therefore, the first flowers on a cluster were collected after GA_3_ treatments, the fourth flowers on a cluster were collected after PP_333_ treatment. After collection, removing the petals and sepals, then the calyx abscission zone tissues, containing calyx tube and a few layers of abscission zone cells on the proximal side of the separation line and adjacent cells, were manually dissected from the calyx tube samples. The collected flowers on the first, fifth and ninth days of the late bloom stage with GA_3_ treatment will be referred to as C_1, C_5, C_9, respectively. The collected flowers on the first, fifth and ninth days of the late bloom stage with PP_333_ treatment will be referred to as T_1, T_5, T_9, respectively. The calyx abscission zone (AZ) tissues were immediately frozen in liquid nitrogen and stored at − 80 °C until use. A Korla fragrant pear with calyx persistence and calyx abscission are shown in Additional file 1. The calyx AZ tissues samples are shown in Additional file 2.

### RNA extraction and quality assessment

Total RNA was extracted from the calyx AZ tissue using EASYspin Plant microRNA Kit (Aidlab, Beijing, China) following the manufacturer’s instructions. The total RNA samples from the six different AZ tissues in the same group were pooled together based on an equal RNA quantity. The RNA degradation and contamination was monitored on 1% agarose gels. RNA purity was checked using the NanoPhotometer® spectrophotometer (IMPLEN, CA, USA). The RNA concentration was measured using Qubit® RNA Assay Kit in Qubit® 2.0 Flurometer (Life Technologies, CA, USA). The RNA was assessed further for RNA integrity using the RNA Nano 6000 Assay Kit of the Agilent Bioanalyzer 2100 system (Agilent Technologies, CA, USA).

### Transcriptome sequencing

The AZ tissues mRNAs (transcripts) of Korla fragrant pear flower were analyzed using the mRNA-seq technique. For six cDNA library constructions, a total amount of 1.5 μg RNA per group was used as input material for the RNA sample preparations. The library for sequencing was generated using a NEBNext® Ultra™ RNA Library Prep Kit for Illumina® (NEB, USA) and index codes were added to attribute sequences to each sample. Transcriptome sequencing was carried out on an Illumina HiSeq 2500 platform and paired-end raw reads were generated. After removing reads containing adapter, reads containing ploy-N, and low quality reads, the remaining clean reads were assembled using Trinity softwareas described for de novo transcriptome assembly without a reference genome [27]. Illumina sequencing was performed at Novogene, Beijing, China.

To understand the function of the genes, gene function was annotated based on the following databases: SwissProt (A manually annotated and reviewed protein sequence database); NR (NCBI non-redundant protein sequences); NT (NCBI non-redundant nucleotide sequences); PFAM (Protein family); KO (KEGG Ortholog database); GO (Gene Ontology); and KOG/COG (Clusters of Orthologous Groups of proteins/euKaryotic Ortholog Groups).

### Differential expressed genes (DEGs) analysis

The expression level of each transcript was measured as the number of clean reads mapped to its reference sequence. The mapped clean read number was normalized to RPKM (expected number of Fragments Per Kilobase of transcript sequence per Millions base pairs sequenced) with RSEM [28]. Differential expression analysis of two groups was performed using the DEGSeq R package. Q value was used to adjust the *p* value [29]. q ≤ 0.005 & |log2 (fold change)| > 1 was set as the threshold for DEGs selection. DEGs were further employed to KEGG (Kyoto Encyclopedia of Genes and Genomes) pathway. We used KOBAS [30] software to test the statistical enrichment of DEGs in KEGG pathways.

### Small RNAs sequencing

A total amount of 3 μg total RNA per group was used as input material for the small RNA library. Sequencing libraries were generated using NEBNext® Multiplex Small RNA Library Prep Set for Illumina® (NEB, USA.) following manufacturer’s recommended protocol and index codes were added to attribute sequences to each sample. Then the libraries were sequenced by Illumina Hiseq 2500/2000 platform and 50 bp single-end reads were generated. Raw data of fastq format were firstly processed through custom perl and python scripts. Clean data were obtained by removing reads containing ploy-N, with 5′ adapter contaminants, without 3′ adapter or the insert tag, containing ploy A or T or G or C and low quality reads from raw data. Then sRNAs were mapped to the *Pyrus sinkiangensis* Yu transctriptome using the Bowtie method [31] without mismatch. Tags originating from protein-coding genes, repeat sequences, rRNA, tRNA, snRNA, and snoRNA, small RNA tags were mapped to RepeatMasker. Rfam database were removed from the clean datas.

Subsequently, unique sequences 18–30 nt in length were mapped to specific species precursors in miRBase 22.0 by modified software mirdeep2 [32] and srna-tools-clito (http://srna-tools.cmp.uea.ac.uk/) to identify known miRNAs. The available software miREvo [33] and mirdeep2 were integrated to predict novel miRNAs through exploring the secondary structure, the Dicer cleavage site and the minimum free energy of the small RNA tags unannotated in the former steps.

### Analysis of differential expressed miRNAs (DEMs)

The expression levels of miRNAs were normalized by TPM [34]: Normalized expression = mapped readcount/Total reads × 1,000,000. Analyses on the DEMs between two groups was carried out by DEGSeq (2010) R package [35]. The significance threshold was set to be q ≤ 0.01 & |log2 (fold change)| > 1 in this test.

### Targets prediction and function annotation of DEMs

The target genes of DEMs were predicted by psRNATarget (http://plantgrn.noble.org/psRNATarget/?function = 3) (maximum expection value = 5.0). For the convenience of description, “target genes of DEMs” is referred to as “candidate target gene”.

### Integrated analysis of DEGs and DEMs

In order to define all the possible miRNA-mRNA interactions, including positive and negative relationships between miRNA and mRNA expression, we used Cytoscape 3.2.0 to construct the miRNA-mRNA regulatory network. Integration of miRNA-seq with mRNA-seq was completed by integrating candidate target genes and DEGs. For the convenience of description, the intersection of “candidate target gene” and “DEG” will be referred to as “differential target gene”. KEGG enrichment analysis was performed to help elucidate the biological functions and critical signal pathways of differential target genes.

### Quantitative real-time PCR (qRT-PCR) validation

To validate the sequencing data, qRT-PCR was performed to detect the expression patterns of DEMs and DEGs among each group. The expression profiles of 8 mature DEMs and 10 DEGs among the miRNA-mRNA interaction network were validated. Total RNA samples were the same as the small RNAs sequencing samples. Then total RNA was polyadenylated and cDNA was generated using 2 μL of total RNA by Mir-X miRNA First-Strand Synthesis Kit (Takara, Beijing, China). Gene-specific primers, miRNA-specific forward primers and universal reverse primer were designed using Primer Premier 5.0. QRT-PCR was performed on a CFX 96 Touch RT-PCR detection system (Bio-Rad, USA) with SYBR Green Real-time PCR Master Mix (Toyobo, Osaka, Japan). The mRNAs and miRNA qRT-PCR method was according to previous studies [20, 21]. U6 and Actin were used as the internal controls for miRNAs and mRNAs, respectively. The expression level was calculated by 2^−ΔΔCT^ method [36]. Pearson correlation coefficient was computed for each miRNA/mRNA expression level and mRNA-Seq/miRNA-Seq data. Additional file 3 shows the sequences of the above primers.

## Result

### Analysis of transcriptome sequencing

**Fig. 1:**
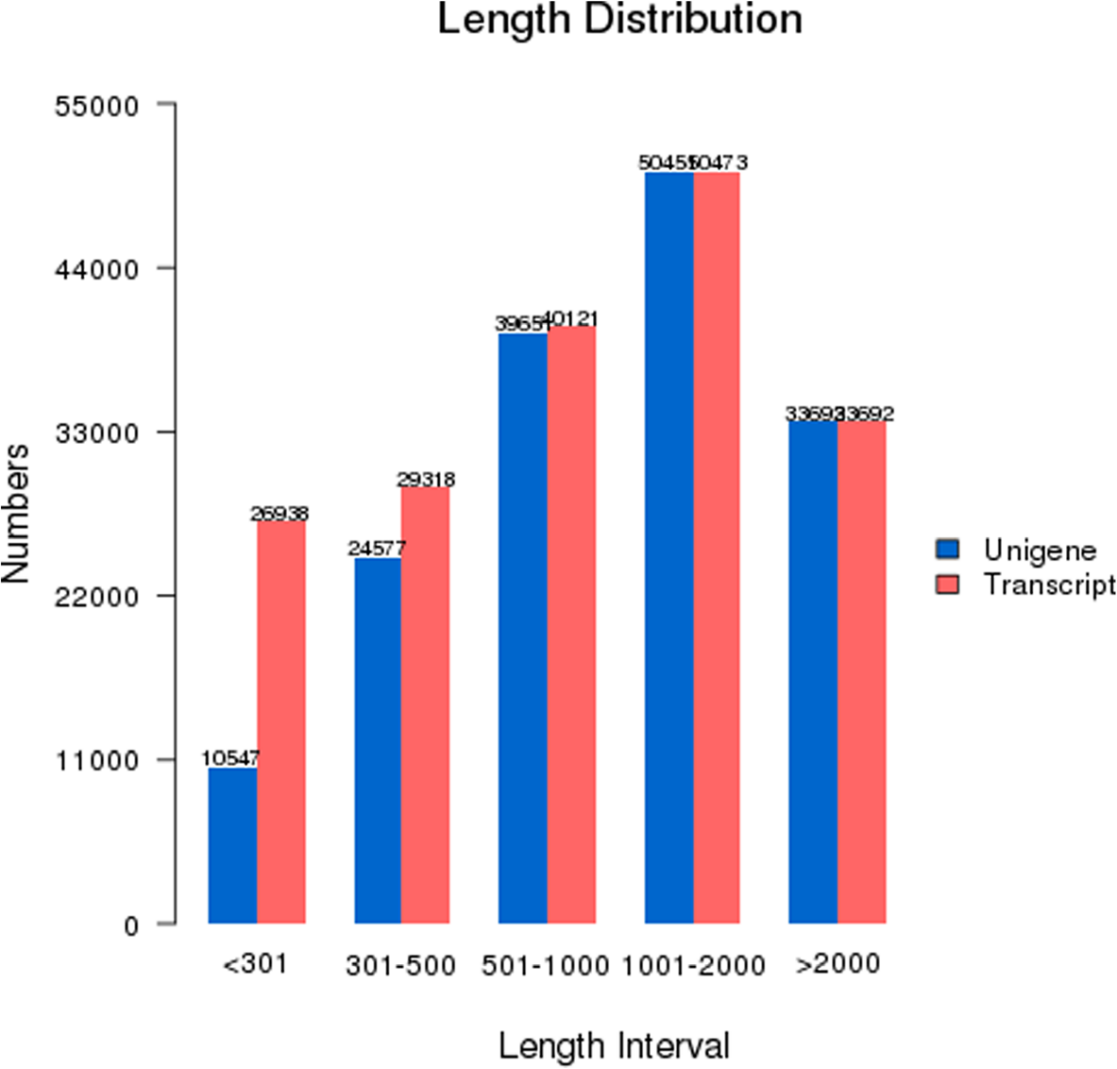
Distribution of the assembled genes and transcript length

Six cDNA libraries representing the calyx AZ tissues of Korla fragrant pear in the calyx persistence group (C_1, C_5, C_9) and those in the calyx abscission group (T_1, T_5, T_9) were constructed with total RNA and subjected to Illumina sequencing. Overviews of the sequencing and assembly results for the calyx persistence group and calyx persistence group are shown in Table 1. After removing the low quality raw reads, 308,085,228 clean reads remained. Through the Trinity de novo assembly method, 158,918 non-redundant genes were obtained, and 180,542 transcripts were achieved with an N50 of 1835 bp and an N90 of 566 bp (Table 2). The length distribution of genes and transcripts larger than 300 bp are shown in Fig. 1.

**Table 1:** Summary of the sequence analyses

Sample	Raw Data	Clean Data	Clean Bases	Error(%)	Q30(%)	Q20(%)	GC(%)
C_1	52,686,688	51,228,474	7.68G	0.03	93.76	97.82	47.42
C_5	53,814,950	52,655,664	7.9G	0.03	93.55	97.74	47.40
C_9	52,368,624	50,631,012	7.59G	0.03	93.69	97.79	47.07
T_1	52,167,050	50,839,792	7.63G	0.03	93.64	97.8	47.02
T_5	52,710,758	51,367,402	7.71G	0.03	93.59	97.74	46.91
T_9	52,539,880	51,362,884	7.7G	0.03	93.81	97.84	46.82
Summary	316,287,950	308,085,228	46.21G

**Table 2:** Assembly statistics of reads

	Min Length	Mean Length	Median Length	Max Length	N50	N90	Total Nucleotides	All
Gene	201	1326	1075	15,403	1875	661	210,741,731	158,918
Transcript	201	1200	908	15,403	1835	566	216,725,773	180,542

### Functional annotation and classification

**Fig. 2:**
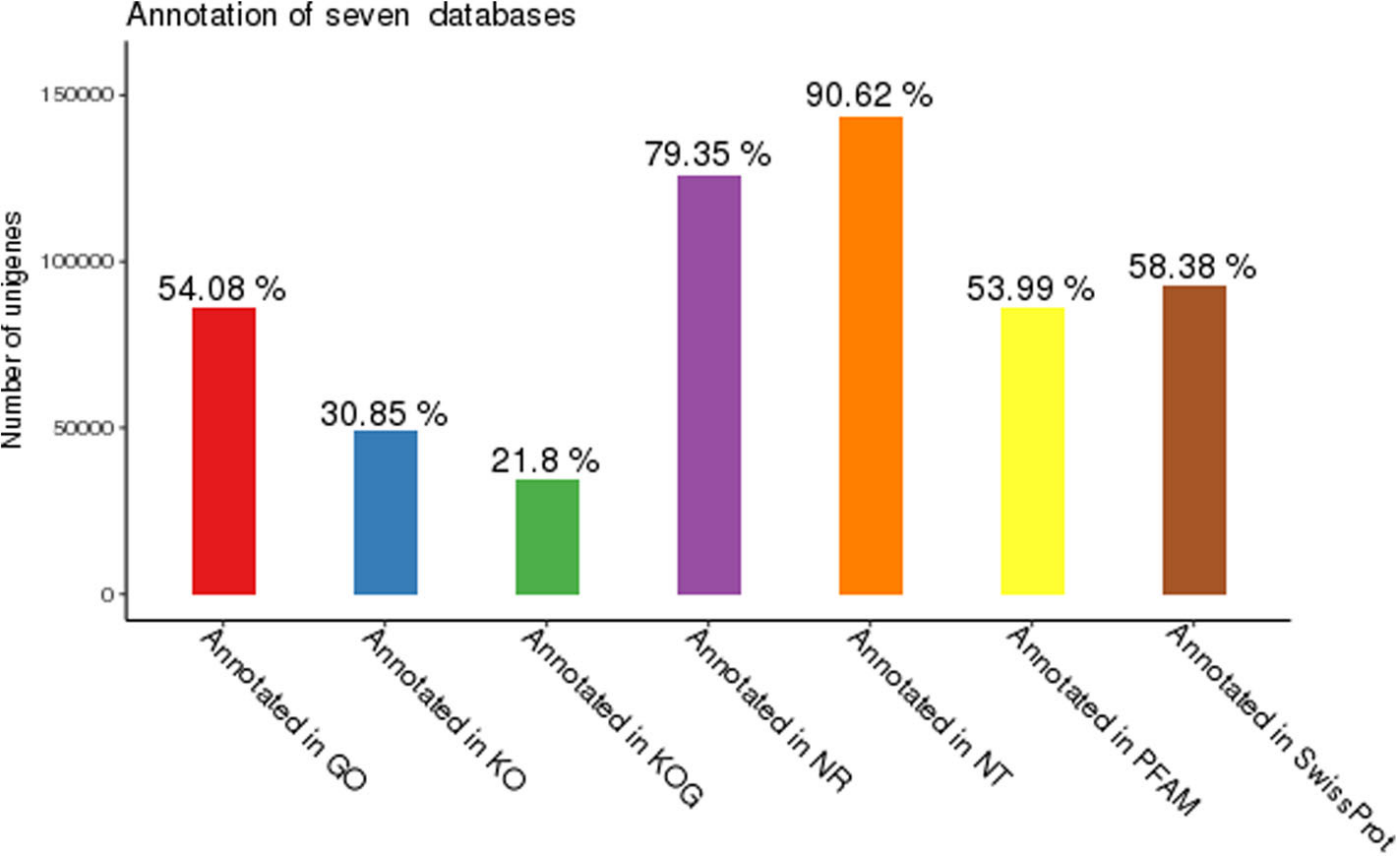
Gene annotation

All the 158,918 assembled genes had significant matches in NR, NT, KO, SwissPort, PFAM, GO, and KOG databases, with the number of genes 126,102 (79.35%), 144,025 (90.62%), 49,033 (30.85%), 92,788 (58.38%), 85,811 (53.99%), 85,952 (54.08%), and 34,650 (21.8%), respectively (Fig. 2).

**Fig. 3:**
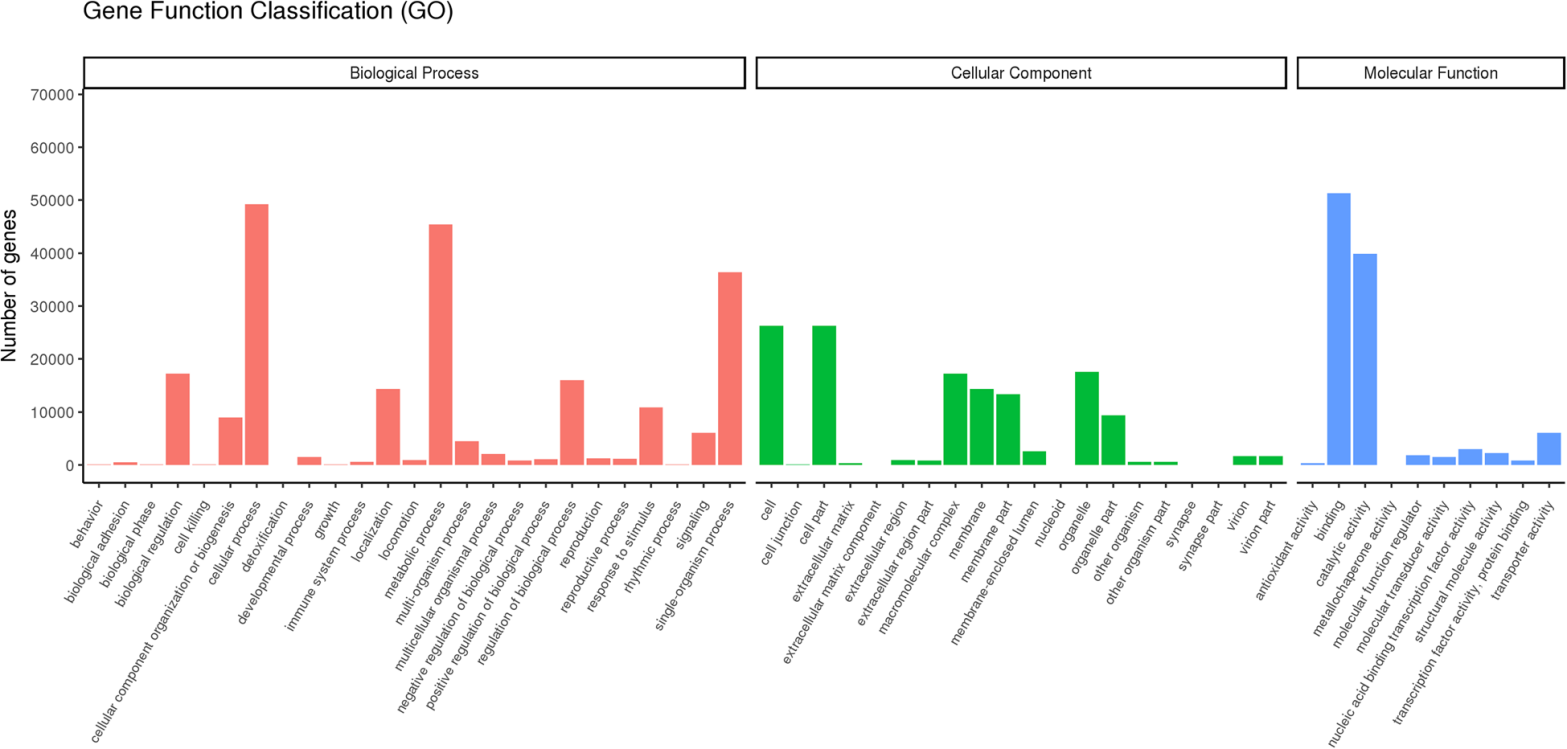
GO categorization of unigenes

For GO biological processes analysis, 85,952 genes were classified into 55 subcategories (Fig. 3). Genes involved in “cellular process” (49212), “single-organism process” (36400) and “metabolic process” (45402) groups were highly represented in the biological process category. Among the cellular components, “cell” (26268) was the most commonly represented, followed by “cell part” (26265) and “organelle” (17538). In the molecular function category, a significant proportion of clusters were assigned to “binding” (51256) and “catalytic activity” (39876).

**Fig. 4:**
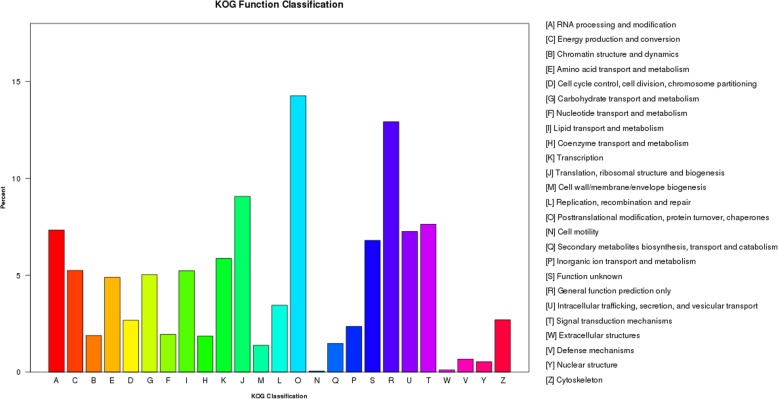
KOG annotation of putative proteins

To classify orthologous gene products, 34,650 genes were subdivided into 25 KOG classifications. Among them, the cluster of “Posttranslational modification, protein turnover, chaperones” (4944) represented the largest group, followed by “General function prediction only” (4480), “Translation, ribosomal structure and biogenesis” (3143), and “Signal transduction mechanisms” (2644). “Extracellular structures” (38) and “Cell motility” (19) were the smallest group (Fig. 4).

**Fig. 5:**
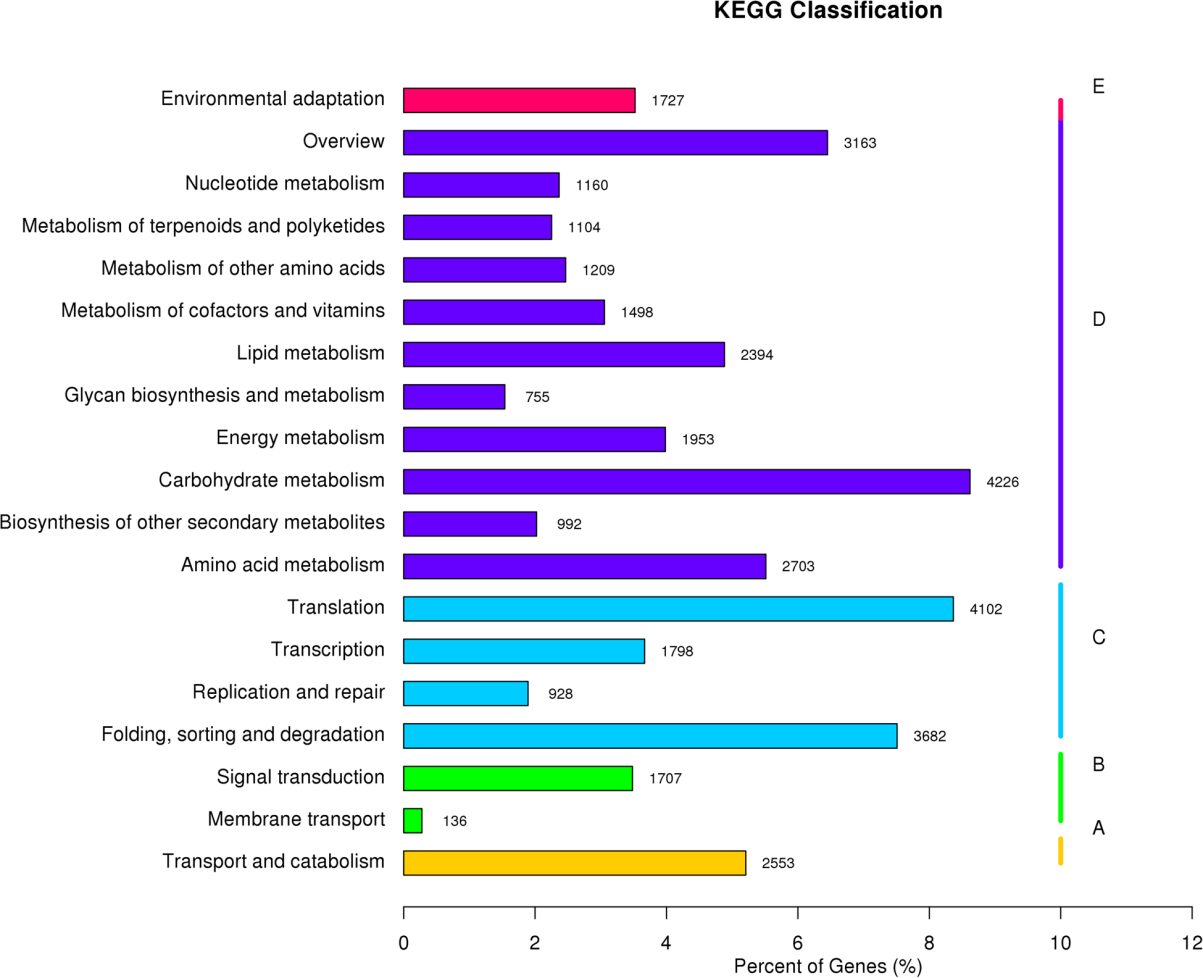
KEGG annotation of putative proteins

The KEGG analysis found that 49,033 genes were classified into 19 pathway categories. The most significantly enriched pathways were “Carbohydrate metabolism” (4226), “Translation” (4102), “Folding, sorting and degradation” (3682), “Overview” (3163), and “Amino acid metabolism” (2703) (Fig. 5).

### DEGs in AZ tissue between calyx persistence group and calyx abscission group

**Fig. 6:**
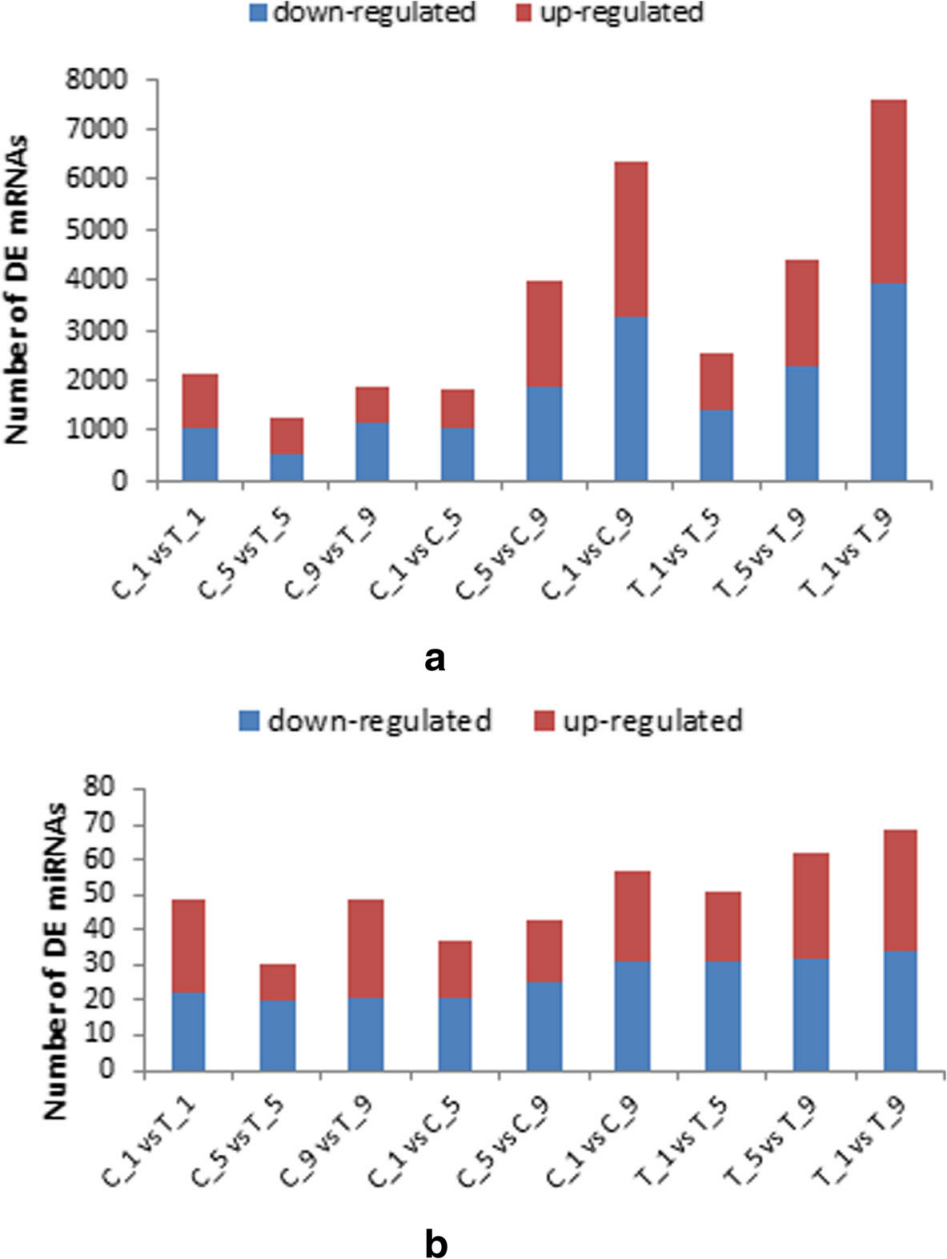
mRNAs and miRNA differentially expressed between different libraries. **a**: mRNAs between different libraries. **b**: miRNAs between different libraries. Up-regulated (red) and down-regulated (blue) mRNAs and miRNA were quantified. The results of 9 comparisons are shown

The analysis of DEGs revealed a significant difference in AZ tissues between calyx persistence group and calyx abscission group. We compared the calyx persistence group with the calyx abscission group, two treatments during the same stage, and the same treatment during different stages, so that 9 pairs of comparisons were implemented, including (i) C_1 vs T_1; (ii) C_5 vs T_5; (iii) C_9 vs T_9; (iv) C_1 vs C_5; (v) C_5 vs C_9; (vi) C_1 vs C_9; (vii) T_1 vs T_5; (viii) T_5 vs T_9; and (ix) T_1 vs T_9. When calyx persistence groups compared with calyx abscission groups during the same stage, 2124, 1280 and 1845 DGEs were identified in C_1 vs T_1, C_5 vs T_5 and C_9 vs T_9, respectively; When C_1 compared with C_5 and C_9, 1835 and 6366 DEGs were screened respectively, and 3991 DEGs were obtained from the comparison between C_5 and C_9; When T_1 compared with the T_5 and T_9, respectively, 2549 and 7577 DEGs were obtained, and 4386 DEGs were obtained from the comparison between T_5 and T_9 (Fig. 6a).

### Pathway enrichment analysis of DEGs

**Fig. 7:**
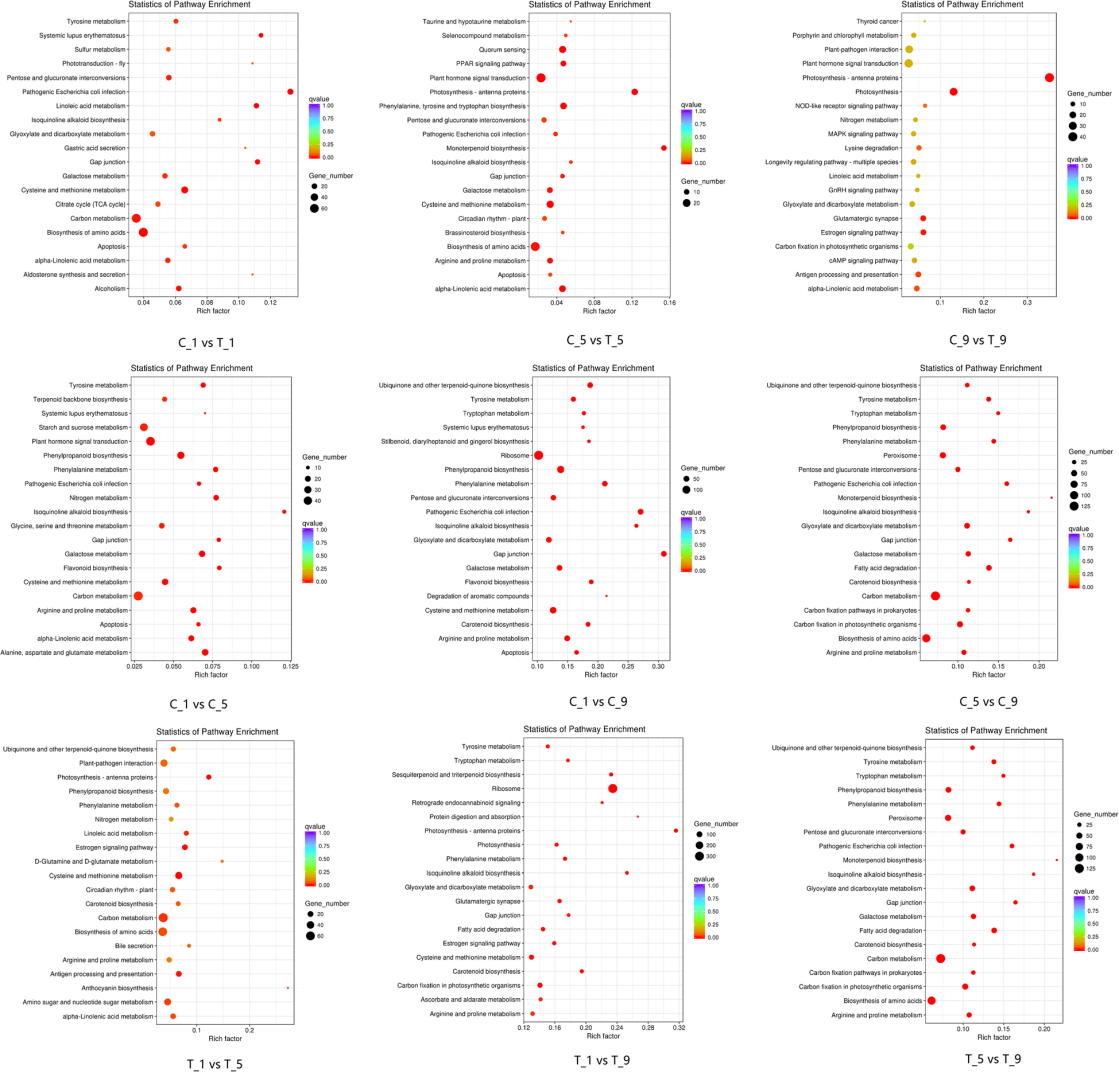
The top 20 KEGG pathways enrichment of DEGs. The x-axis indicates the rich factor and the y-axis indicates the pathway names

To further investigate the function of DEGs during calyx abscission, significantly enriched KEGG pathways were analyzed. The DEGs which were screened out with calyx persistence group as a control and compared with calyx abscission group, respectively, were mainly enriched in alpha-Linolenic acid metabolism, plant hormone signal transduction and photosynthesis. The DEGs in C_1 vs C_5, C_1 vs C_9, and C_5 vs C_9 were mainly enriched in plant hormone signal transduction, in addition to the galactose metabolism and carotenoid biosynthesis. The DEGs in T_1 vs T_5, T_1 vs T_9, and T_5 vs T_9 were mainly enriched in ribosome, photosynthesis and cysteine and methionine metabolism (Fig. 7). These results imply that the genes involved in these pathways may play crucial roles in calyx abscission.

### Analysis of miRNA sequencing

**Fig. 8:**
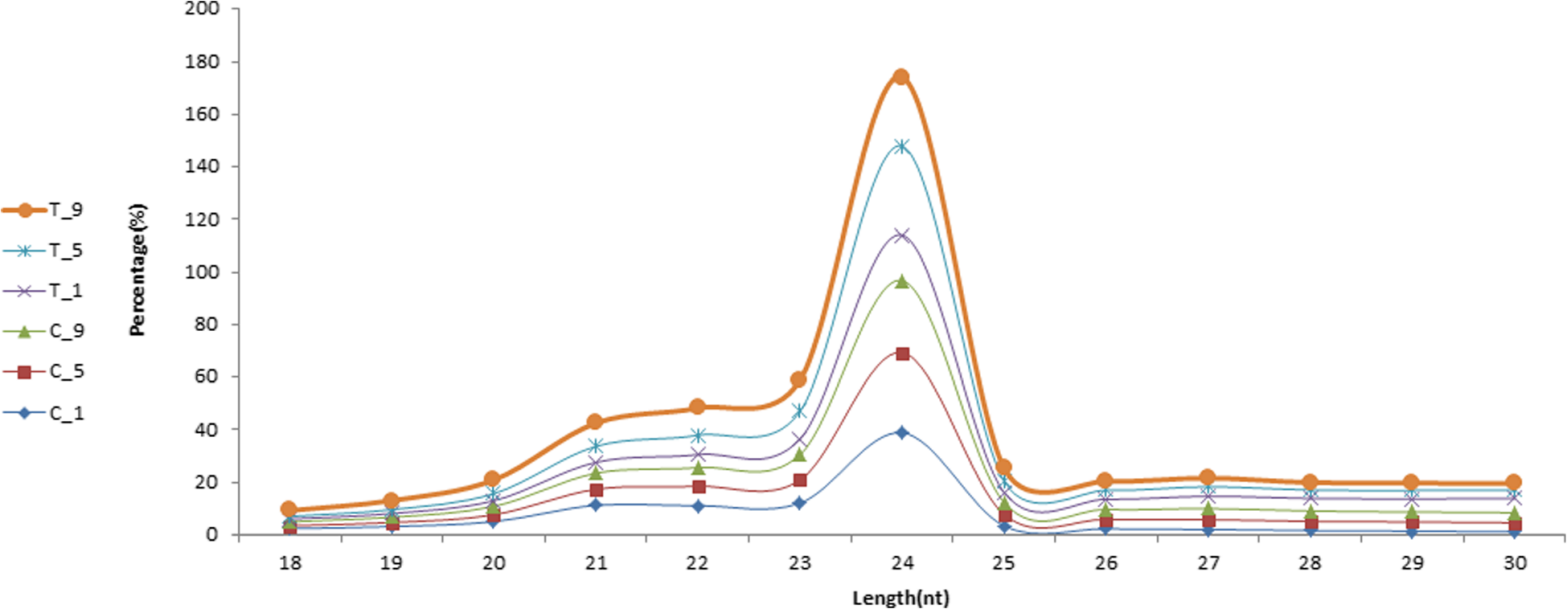
Length distribution of sRNAs in the six libraries

A total of 95,888,963 raw reads were obtained from the six libraries. After removing the low quality reads and adapters, 14,816,133, 13,952,977, 14,223,817, 14,122,942, 19,289,704, 14,150,546 clean reads were obtained in C_1, C_5, C_9, T_1, T_5, and T_9 samples, respectively. An overview of small RNA classification annotation result statistics is shown in Table 3. Clean reads with a length of 18 to 30 nt were selected for further analysis. Figure 8 shows the length distribution of the sRNAs in the six libraries. The length distribution of clean reads showed that most of the reads were between 23 and 25 nt in length, and read counts with 24 nt were highest.

**Table 3:** Summary of small RNA sequencing and annotation in the six libraries

	C_1	C_5	C_9	T_1	T_5	T_9
Raw reads	15,286,088	14,558,545	15,081,645	16,628,582	19,808,737	14,525,366
Clean reads	14,816,133	13,952,977	14,223,817	14,122,942	19,289,704	14,150,546
sRNA reads with 18–30 nt	13,297,814	11,786,437	11,412,245	9,257,732	17,475,944	12,527,738
Mapped sRNA reads	6,624,449	7,232,363	7,210,889	6,924,934	9,807,723	7,212,813
Known miRNA	105,499	38,469	28,187	15,050	74,146	36,416
Novel miRNA	77,212	38,278	38,410	27,118	69,111	55,420
rRNA	796,814	1,041,300	1,150,503	1,136,897	1,328,107	1,006,974
tRNA,	1	0	0	0	0	0
snRNA	6057	7915	8336	6040	8069	17,190
snoRNA,	23,253	25,748	33,010	25,689	45,378	36,739
ta-siRNA,	60,908	29,424	27,270	12,129	47,678	32,416
Others	5,554,705	6,051,229	5,925,173	5,702,011	8,235,234	6,027,658

### Identification of conserved and novel miRNAs

In total, we identified 48 conserved miRNAs belonging to 24 miRNA families, and 84 predicted novel miRNAs in the six small RNA libraries (Additional file 4: Table S2). Details regarding family member numbers of conserved miRNA are summarized in Additional file 5: Table S3. A total of 18 conserved miRNA families contained more than one member.

### DEMs in AZ tissue between calyx persistence group and calyx abscission group

In the miRNA-sequencing study, we obtained 49, 30 and 49 miRNAs differentially expressed in C_1 and T_1, C_5 vs T_5 and C_9 vs T_9, respectively. In addition, 37, 57 and 43 DEMs were identified in C_1 vs C_5, C_1 vs C_9 and C_5 vs C_9, respectively. And there were 51, 69 and 62 DEMs in T_1 vs T_5, T_1 vs T_9 and T_5 vs T_9, respectively (Fig. 6b). This trend was similar to the number of DEGs.

### Pathway analysis of candidate target gene

To better understand the functions of the DEMs identified, we predicted the potential target genes of these miRNAs. There were 20,744 target genes were predicted (Additional file 6: Table S4). Through the KEGG enrichment analysis, we found 45 KEGG pathways significantly (q value≤0.05) related with genes targeted by miRNAs (Additional file 7: Table S5). A lot of pathways were involved in carbohydrate metabolism and amino acid metabolism.

### Integrated analysis of DEGs and DEMs

To explore miRNA and mRNA regulatory networks in AZ tissues, the expression profiles of miRNA and mRNA were combined for further analysis. There are a total of 2587 miRNA-mRNA pairs among the six treatment groups, with both positive and negative correlation identified. In total, we obtained 1206 differential target genes and 114 miRNAs through integrated analysis. Compared calyx persistence groups with calyx abscission groups during the same stage, 145, 56 and 150 mRNA-miRNA pairs were obtained in C_1 vs T_1, C_5 vs T_5 and C_9 vs T_9, respectively; When C_1 compared with C_5 and C_9, 90 and 506 mRNA-miRNA pairs were screened respectively, and 255 mRNA-miRNA pairs were obtained from the comparison between C_5 and C_9; When T_1 compared with the T_5 and T_9, respectively, 206 and 796 mRNA-miRNA pairs were obtained, and 383 mRNA-miRNA pairs were obtained from the comparison between T_5 and T_9 (Additional file 8). The majority of mRNA-miRNA pairs present a negatively correlated expression pattern. Most miRNAs had more than one possible target gene, while different miRNAs could regulate the same targets. For instance, in C_1 vs T_1, psi-miR827 was the regulator of Cluster-9706.71346, Cluster-9706.109338 and Cluster-9706.121318, whereas psi-miR171a-3p and psi-miR171b-3p could regulate the expression of Cluster-9706.61759.

### KEGG enrichment analysis of differential target genes

**Fig. 9:**
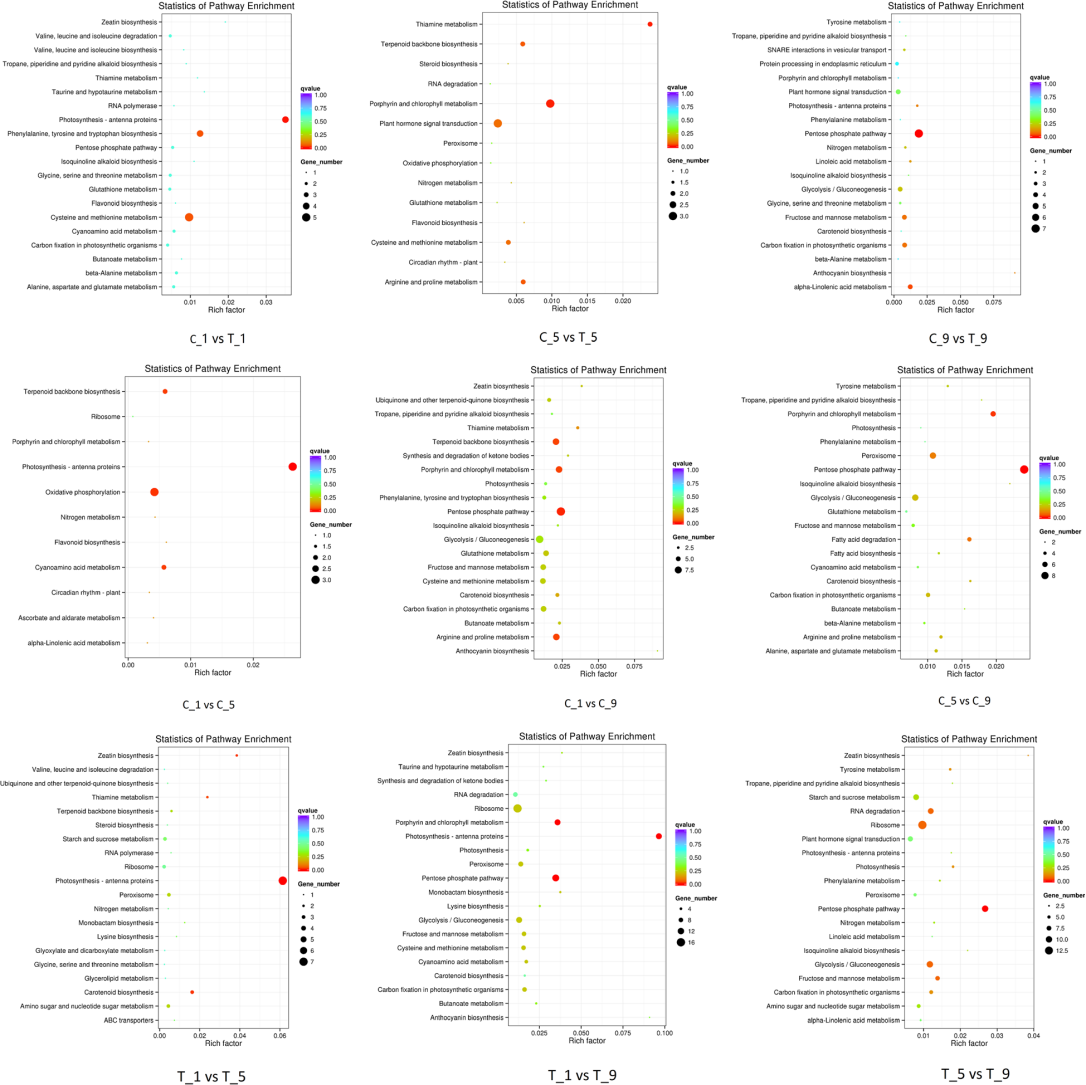
The top 20 KEGG pathways enrichment of differential target genes. The x-axis indicates the rich factor and the y-axis indicates the pathway names

To better understand the biological functions of these differential target genes, we performed the KEGG pathway analyses. The differential target genes in C_1 vs T_1 were mainly enriched in photosynthesis - antenna proteins, cysteine and methionine metabolism, and phenylalanine, tyrosine and tryptophan biosynthesis; differential target genes in C_5 vs T_5 were mainly enriched in plant hormone signal transduction, porphyrin and chlorophyll metabolism, and cysteine and methionine metabolism; In C_9 vs T_9, differential target genes were mainly enriched in pentose phosphate pathway, in addition to the alpha-Linolenic acid metabolism and carbon fixation in photosynthetic organisms. The differential target genes in C_1 vs C_5, C_1 vs C_9, and C_5 vs C_9 were mainly enriched in photosynthesis - antenna proteins, pentose phosphate pathway, and porphyrin and chlorophyll metabolism. The differential target genes in T_1 vs T_5, T_1 vs T_9, and T_5 vs T_9 were mainly enriched in photosynthesis - antenna proteins, carotenoid biosynthesis, and pentose phosphate pathway (Fig. 9, Additional file 9).

To further explain the possible mechanism involved in calyx abscission, miRNA-mRNA analysis narrowed down to key genes and significantly enriched pathways identified by KEGG analysis. Combined with the KEGG enrichment analysis of DEGs, we conclude that some important metabolic pathways may be associated with calyx abscission, including terpenoid backbone biosynthesis, photosynthesis - antenna proteins, photosynthesis, porphyrin and chlorophyll metabolism, carotenoid biosynthesis, zeatin biosynthesis and plant hormone signal transduction. In addition, we obtained some key genes from miRNA-mRNA pairs for their potential roles in calyx abscission according to their annotations and their potential relationship with abscission-responsive miRNAs, including protein phosphatase 2C (psi-miR394a-*HAB1*), cellulose synthase-like protein D3 (psi-miR827-*CSLD3*), beta-galactosidase (psi-miR858b-β-galactosidase), zinc finger protein (psi-miR319a-*ZAT12*), abscisic acid 8′-hydroxylase 1 (psi-miR396a-5p-*CYP707A1*), Laccase-7 (psi-miR397a-*LAC7*), polygalacturonase (psi-miR396b-3p-*GSVIVT00026920001*), receptor-like protein kinase (psi-miR396a-5p-*HERK1*) and auxin response factor (psi-miR160a-3p-*ARF6*, psi-miR167d-*ARF18*, psi-miR167a-5p-*ARF25*), etc.

### Confirmation of DEGs and DEMs by qRT-PCR

**Fig. 10:**
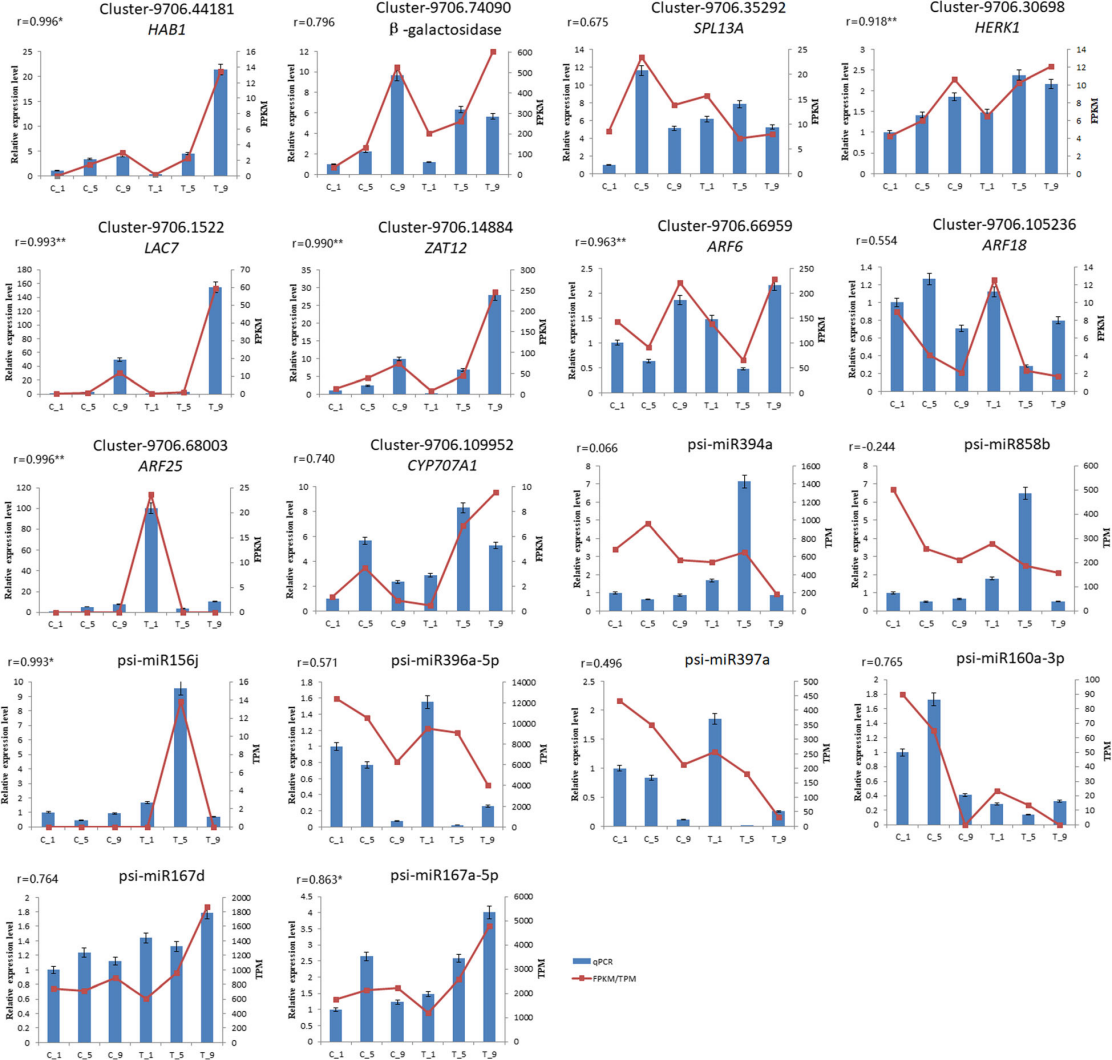
Real-time PCR validation of several DEGs and DEMs in miRNA-mRNA pairs. The x-axis represents RNA names, the left y-axis represents relative expression level, and right y-axis represents FPKM/TPM. Blue bars represent data yielded by qRT-PCR, and red points represent data obtained by RNA sequencing, ‘r’ represents Pearson correlation coefficient, *: correlation is significant at 0.05 level. **: correlation is significant at 0.01 level

The expression profiles of 8 DEMs (psi-miR394a, psi-miR858b, psi-miR397a, psi-miR396a-5p, psi-miR156j, psi-miR160a-3p, psi-miR167d, psi-miR167a-5p) and 10 DEGs {*HAB1*(Cluster-9706.44181), Beta-galactosidase (Cluster-9706.74090), *HERK1* (Cluster-9706-30,698), *LAC7* (Cluster-9706.1522), *ZAT12* (Cluster-9706.14884), *SPL13A* (Cluster-9706.35292), *ARF6* (Cluster-9706.66959), *ARF18* (Cluster-9706.105236), *ARF25* (Cluster-9706.68003), *CYP707A1* (Cluster-9706.109952)} among the mRNA-miRNA interaction network were further validated using qRT-PCR (Fig. 10). The results of qRT-PCR revealed that most of these mRNAs/miRNAs share the similar expression tendencies with those from mRNA-Seq/miRNA-Seq data, and Pearson correlation also showed that most of the miRNAs/mRNA expression levels were strongly correlated with mRNA-Seq/miRNA-Seq data, which might partially validate the reliability of our sequence data and our findings in the present study.

## Discussion

In the present study, we simultaneously analysis mRNA and miRNA profiles and construct miRNA-mRNA regulatory networks to enhance our understanding of molecular mechanisms of calyx persistence. This is the first detailed information regarding parallel mRNA and miRNA expression about calyx abscission in Korla fragrant pear. By integrated analysis, we obtained the complete set of miRNAs/mRNAs associated with calyx abscission, their interactions, and regulatory pathway.

### Effect of flower development on calyx persistence of Korla fragrant pear

Whether in the C_1/C_5, C_5/C_9, C_1/C_9 or in T_1/T_5, T_5/T_9, T_1/T_9, the number of DEGs are gradually increased. He et al. found that whether calyx of the most young fruit was dropped or not can be seen at 8 days after full bloom, that is, calyx tube abscission symptoms (yellow loop in abscission zone) were observed (Additional file 1: Figure S1) [37]. In Fig. 6, the number of DEGs between T1/T9 and T5/T9 is much higher than the number of DEGs between T1/T5, it could be considered that a large number of gene associated with calyx abscission expressed on the 5–9 day of the late bloom stage after treatment with PP_333_.

### Pathway analysis of differential target genes related to calyx abscission

Trans-Zeatin-riboside (ZR) is a natural cytokinin. Plant endogenous hormones such as Indole-3-acetic acid (IAA), abscisic acid (ABA), GA_3_ and ZR are involved in the regulation of the calyx persistence process in pear. The high content of IAA, GA_3_ and ZR in the calyx and the low content of ABA are one of the reasons for the persistence of pear fruit [12]. Terpenoids are a very important secondary metabolite in plants. Some terpenoids, such as gibberellin, indoleacetic acid and other plant hormones, play an important role in plant growth and development. Carotenoids and chlorophyll are involved in plant photosynthesis [38]. In plants, carotenoids are mainly found in chloroplasts and colored bodies of flowers and fruits. It is involved in light absorption and photomorphogenesis in photosynthesis [39, 40], and also participate in plant responses to external stimuli [41]. Carotenoids can also be used as precursors of plant hormones (such as abscisic acid) to participate in their anabolic processes [42].The reduction of photosynthesis plays an important role in the process of flower and fruit abscission [43]. Our study found photosynthesis - antenna proteins and photosynthesis pathway was significantly enriched. Other pathway related to photosynthesis such as porphyrin and chlorophyll metabolism and carotenoid biosynthesis were also significantly enriched, indicating that photosynthesis plays an important role in the process of calyx abscission. Plant hormones can regulate the plant growth and development as well as various physiological metabolic processes. Almost all hormones can affect organ abscission, such as ethylene, auxins, abscisic acid, gibberellin and jasmonic acid. Studies have confirmed that ethylene and auxin is the dominant hormone that regulates organ senescence and abscission [44]. In present study, zeatin biosynthesis, terpenoid backbone biosynthesis, carotenoid biosynthesis, photosynthesis and plant hormone signal transduction pathways were significantly enriched. It is indicated that these pathways play an important role in the calyx abscission.

### Functional analysis of miRNA-mRNA pairs

We obtained 1206 differential target genes and 114 miRNAs by combining small RNA sequencing and transcriptome data. These differential target genes represent the target gene group with differential expression regulated by DEMs in AZ tissue after calyx abscission/persistence treatment. This gene group was constructed based on the regulatory relationship between miRNA and mRNA, reflecting the abnormal changes of genes caused by changes in miRNA expression after calyx abscission/persistence treatment. Therefore, these miRNA-mRNA pairs are very potentially involved in calyx abscission. Here, we focus on the known miRNAs and their corresponding differential target genes. We obtained 517 differential target genes and 41 known miRNAs through integrated analysis (Additional file 10).

Laccase (*LAC*) gene family has been reported to be involved in lignin biosynthesis. In *Arabidopsis thaliana*, oxidative polymerization of flavonoid and biosynthesis of lignin have been demonstrated to be catalyzed by laccase-15 [45]. Lignin is an important macromolecular organic material. The content of lignin is only lower than that of cellulose inside plant [46]. Lignin and cellulose, hemicellulose are the main components of the plant skeleton. Genes related to the formation and degradation of lignin has been reported to be involved in organ abscission [47]. Besides, 4-coumarate-CoA ligase plays an important role in the synthesis of lignin [48]. By integrated analysis, we found Laccase-7 (*LAC-7*) was targeted by psi-miR397a, which was significantly differentially expressed in C_1 vs C_9, C_9 vs T_9, T_5 vs T_9 and T_1 vs T_9. In *Populus trichocarpa*, miR397a-*LAC* pair is involved in the formation of lignin [49]. QRT-PCR also indicated that the expression of miR397a and *LAC7* displayed opposite results and was significantly differentially expressed in C_9 vs T_9 (Fig. 10). 4-coumarate--CoA ligase-like 5 (*4CLL5*) was targeted by psi-miR396b-5p and psi-miR396a-5p. According to a previous study, miR396 is involved in flower, leaves and fruits development, hence psi-miR396 presumably regulates *4CLL5* to promote an increase in synthesis of lignin to suppress the calyx abscission [37]. Cellulase is a cell wall hydrolase that plays an important role in the organ abscission process. It degrades cellulose and hemicellulose and is the main constituents of the cell wall [50]. Cellulose synthase-like protein D3 (*CSLD3*) was predicted to be targeted by psi-miR827. The expression level of psi-miR827 was up-regulated in C_9 vs T_9, whereas *CSLD3* was down-regulated, which suggests that the down-regulation of *CSLD3* plays important roles in regulating calyx persistence. E3 ubiquitin-protein ligase (*KEG*) has been shown to play an important role in hormone regulation, photomorphogenesis, flower development, senescence, and pathogen defense in plant [51]. In this study, *KEG* was targeted by psi-miR171a-3p and psi-miR397a, which was up-regulated in C_1 vs T_1, C_9 vs T_9, C_5 vs C_9, and C_1 vs C_9.

A negative correlation was also uncovered between the expression of psi-miR396b-5p and its target, ABA 8′-hydroxylase 1 (*CYP707A1*). *CYP707A1* is a key enzyme for the oxidative decomposition of ABA, which play a negative regulation role in the accumulation of ABA [52]. Overexpression of *CYP707A1* reduced ABA levels and exhibited an ABA-deficient phenotype [53]. Hormones such as ABA, cytokinin and sorbitol lactone play a key role in apple abscission [54]. Our results showed that psi-miR396b-5p was down-regulated while its target *CYP707A1* was up-regulated in T_5 and T_9. This suggests that the psi-miR396b-5p-*CYP707A1* pair is associated with calyx abscission in that down-regulated psi-miR396b-5p presumably up-regulates *CYP707A1* expression to suppress an increase in ABA expression to promote the calyx abscission. The results, which were confirmed by qRT-PCR, suggested that psi-miR396b-5p and *CYP707A1* are involved in calyx persistence (Fig. 10).

Cell separation is thought to be related to pectin degradation caused by several degrading enzymes, especially polygalacturonase (PG). PGs are the one of cell wall hydrolytic enzyme families that involved in the degradation of pectin in plant cell walls and intercellular layers. It is involved in the plant development such as seed germination, dehiscence, pollen ripening, pollen sac cracking, fruit ripening and organ abscission [55, 56]. In this study, psi-miR396b-3p was down-regulated while its target PG was up-regulated in T_9 relative to T_1. This suggests that the reduced expression of psi-miR396b-3p enhanced the expression of PG that may regulate calyx abscission.

Many researches have proved that protein phosphatase 2C is involved in several life activities, such as plant signal transduction pathways and disease resistance. In recent years, some protein phosphatases have also been reported to be involved in the signal transduction of plant organs abscission [57]. Corbacho et al. [58] found that protein phosphatase may be involved in the regulation of melon fruit abscission. Meir et al. [59] examined transcriptome changes in the tomato flower AZ tissue and found that a protein phosphatase gene can affect the abscission process. These indicate that protein phosphatase 2C may be involved in the regulation of organ abscission. By integrated analysis, psi-miR394a was predicted to target one protein phosphatase 2C gene and three probable protein phosphatase 2C genes in C_9 vs T_9, one protein phosphatase 2C gene and six probable protein phosphatase 2C genes in T_1 vs T_9. The expression level of psi-miR394a was down-regulated in T_5 vs T_9, whereas protein phosphatase 2C (*HAB1*) was up-regulated, which suggests that the up-regulation of *HAB1* plays important roles in calyx abscission (Fig. 10).

Current research indicates that plant receptor-like kinases play an important role in regulating plant growth and development, immune response and cell death [60, 61]. Some receptor-like kinases such as *HAESA*/*HSL2*, *EVR*, *SERK1* and CSJ have been reported to control the abscission of flower organs [62–65]. *IDA*, a gene that may encode a short peptide ligand, affects the final stage of AZ cell separation in the flower organ of *Arabidopsis thaliana* [66]. The leucine-rich repeat receptor-like kinase *HAESA* in Arabidopsis is an *IDA* receptor, which also plays an important role in flower organ abscission. In *Arabidopsis thaliana*, knockout of *HAESA* and its homologous gene *HAESA-LIKE 2* (*HSL2*) can significantly delay the flower organs abscission [67]. Among differential target genes, we found a large number of genes related to receptor-like protein kinase and probable receptor-like protein kinase. There is one gene encoding cysteine-rich receptor-like protein kinase 10 targeted by psi-miR160a-3p and psi-miR171a-3p in C_1 vs T_1 and C_1 vs C_9, respectively, two genes encoding receptor-like cytosolic serine/threonine-protein kinase RBK2 targeted by psi-miR399b in C_5 vs C_9, four receptor-like protein kinase genes in C_1 vs C_9, one receptor protein kinase gene targeted by psi-miR396a-3p in T_1 vs T_5, sixteen receptor-like protein kinase genes in T_5 vs T_9. QRT-PCR showed that the expression of psi-miR396a-5p gradually decreased with PP_333_ treatment, while its target receptor-like protein kinase HERK 1 expression gradually increased (Fig. 10). We speculate that these mRNA-miRNA pairs are very potentially associated with calyx abscission.The beta-galactosidase is involved in cell wall degradation. Wu and Burns [68] identified the sequence encoding β-galactosidase in a search for differentially expressed genes during the abscission process in citrus. Southern blot analysis demonstrated that at least two closely related b-galactosidase genes were associated with fruit abscission. The citrus beta-galactosidase was expressed in the stamens, petals and young fruitlets at full bloom stage, indicating that the beta-galactosidase gene may play a role during abscission as well as early growth and development processes in flowers and fruitlets. We found several genes encoding beta-galactosidase among differential target genes. There are one beta-galactosidase genes in C_1 vs C_5, two beta-galactosidase genes in C_1 vs C_9, and three beta-galactosidase genes in T_1 vs T_9. All of them were targeted by psi-miR858b, which may promote calyx abscission by regulating β-galactosidase expression.

Auxin has the effect of inhibiting organ abscission. In *Arabidopsis thaliana*, the IAA signaling pathway in the abscission layer is necessary to regulate organ abscission [69]. Auxin response factors *ARF1*, *ARF2*, *ARF7*, and *ARF19* genes of *Arabidopsis thaliana* are involved in the regulation of flower organ abscission [70, 71]. Our result showed that *ARF6*, *ARF18*, and *ARF25* were targeted by psi-miR160a-3p, psi-miR156j and psi-miR167d/167a-5p, respectively. Both qRT-PCR and sequencing result showed that these mRNA-miRNA pairs were significantly differentially expressed between calyx persistence tissue and calyx abscission tissue (Fig. 10). In tea plant (*Camellia sinensis*) and *Asparagus officinalis*, ARF was also identified as the target genes of miR160 through 5’RLM-RACE and exhibited a negative correlation [72]. In Trifoliate Orange (*Poncirus trifoliata*), ARF was identified as the target genes of miR167 through 5’RLM-RACE [73]. The nucleotide sequence of miR160 in *Camellia sinensis* and *Asparagus officinalis* are identical to the nucleotide sequence of psi-miR160a-3p. The nucleotide sequence of miR167 in Trifoliate Orange is also same as the nucleotide sequence of psi-miR167a-5p. All these can prove the reliability of our results. Consequently, psi-miR160a-3p, psi-miR167d, psi-miR167a-5p and psi-miR156j may promote calyx abscission by regulating *ARF* expression.

By integrated analysis, we also obtained other miRNA-mRNA pairs that may be related to calyx abscission. We found psi-miR319a targeted zinc finger protein (*ZAT12*) in C_1 vs C_9 and T_1 vs T_9, psi-miR396b-3p targeted B-box zinc finger protein 32 in T_1 vs T_5 and T_1 vs T_9. Cho et al. [74] reported that over-expressed zinger finger protein (*AtZFP2*) can delay flower organ abscission in *Arabidopsis thaliana*. *SPL* may be regulated by psi-miR156j/psi-miR157d, and thus is associated with organ abscission [75, 76]. *NAC100* was predicted to be targeted by psi-miR164a in T_1 vs T_5 and T_1 vs T_9. The expression of genes induced by abscisic acid is regulated by NAC and is highly expressed in the detached areas of olives [77] and apples [78]. In Trifoliate Orange (*Poncirus trifoliata*), SPL transcription factor gene was also verified as a target of ptr-miR156 through 5’RLM-RACE [73]. Jeyaraj et al. [72] confirmed through 5’RLM-RACE that csn-miR164a was predicted to target *NAC100* and exhibited a negative correlation. Li et al. [79] showed that *NAC100* can be regulated by cleavage in the binding region between the 10th and 11th base from 5′ end pairing of Zm-miR164, consistent with Jeyaraj’s reports. These studies support the efficacy of our target prediction analysis. Among differential target genes, we obtained four *SPL* genes in C_5 vs T_5, two *SPL* genes in C_1 vs T_1, four *SPL* in C_1 vs C_5, four *SPL* genes in C_5 vs C_9, two *SPL* genes in C_1 vs C_9, one *SPL* gene in T_1 vs T_5, and seven *SPL* genes in T_1 vs T_9.

Several weaknesses in this study should be noted. First, bioinformatics analysis based on transcriptome sequencing and small RNAs sequencing were performed, and several mRNA and miRNA expression were validated by qRT-PCR. The cleavage site of predicted miRNA targets should be validated through 5’RLM-RACE in the future. Second, given the down-regulation of miRNAs revealed in existing studies, we focused on exploring negative miRNA-mRNA regulatory pairs in our present study. However, our study also found a lot of positive miRNA-mRNA pairs. Further exploration is needed to reveal the positive regulation of miRNAs on genes. Third, we identified 84 novel miRNAs by small RNA sequencing, many novel miRNAs were also associated in the integrated analyze. The authenticity of these novel miRNAs needs further identification.

## Conclusions

In summary, by integrated analysis mRNA and miRNA, we established the network of miRNA-mRNA pairs to learn about precise regulation of miRNA on calyx abscission. There are a total of 2587 miRNA-mRNA pairs among the six treatment groups, with both positive and negative correlation identified. These differential target genes were mainly involved in terpenoid backbone biosynthesis, photosynthesis-antenna proteins, porphyrin and chlorophyll metabolism, carotenoid biosynthesis, zeatin biosynthesis and plant hormone signal transduction. In addition, some key miRNA-mRNA pairs may related to calyx abscission, including protein phosphatase 2C (psi-miR394a-*HAB1*), receptor-like protein kinase (psi-miR396a-5p-*HERK1*), cellulose synthase-like protein D3 (psi-miR827-*CSLD3*), beta-galactosidase (psi-miR858b-β-galactosidase), *SPL*-psi-miR156j/157d, abscisic acid 8′-hydroxylase 1 (psi-miR396a-5p-*CYP707A1*) and auxin response factor (psi-miR160a-3p-*ARF6*, psi-miR167d-*ARF18*, psi-miR167a-5p-*ARF25*) etc., were obtained through integrated analysis. Taken together, although this study cannot completely account for the calyx abscission in Korla fragrant pear, the mRNAs and miRNAs revealed in this study will be helpful in understanding the possible mechanism involved in calyx abscission.

## Additional files

Additional file 1: Flowers with persistent and deciduous calyx of Korla fragrant pear. (1). Flowers with persistent calyx. (2). Flowers with deciduous calyx. The white arrow points to the separation line of the calyx abscission. (3). The ‘a’ indicate flowers with deciduous calyx, and the calyx have fallen off. The ‘b’ indicate flowers with persistent calyx. (DOCX 2415 kb)

Additional file 2: The calyx abscission zone (AZ) tissues samples. a: Sample of Korla fragrant pear flower without petals. b: Sample of calyx abscission zone (AZ) tissues. (DOCX 878 kb)

Additional file 3: The qRT-PCR primers. (DOCX 22 kb)

Additional file 4: Known and novel miRNAs in six Korla fragrant pear libraries. (XLSX 19 kb)

Additional file 5: List of miRNA member in each family in Korla fragrant pear. (XLSX 9 kb)

Additional file 6: Potential target genes of DEMs predicted by psRNATarget. (XLSX 1488 kb)

Additional file 7: KEGG enrichment of candidate target genes. (XLSX 11 kb)

Additional file 8: miRNA-mRNA pairs among the six treatment groups. (PDF 4832 kb)

Additional file 9: DEGs KEGG enriched TOP20. (XLSX 25 kb)

Additional file 10: Known miRNA-mRNA pairs among the six treatment groups. (XLSX 124 kb)

## Abbreviations

ABA: Abscisic acid
C_1: The collected flowers on the first day of the late bloom stage with GA_3_ treatment
C_5: The collected flowers on the fifth day of the late bloom stage with GA3 treatment
C_9: The collected flowers on the ninth day of the late bloom stage with GA_3_ treatment
DEG: Differential expressed genes
DEM: Differential expressed miRNAs
GA_3_: Gibberellin
GO: Gene Ontology
IAA: Indole-3-acetic acid
KEGG: Kyoto Encyclopedia of Genes and Genomes
KO: KEGG Ortholog database
KOG/COG: Clusters of Orthologous Groups of proteins
Nr: NCBI non-redundant protein sequences
Nt: NCBI non-redundant nucleotide sequences
Pfam: Protein family
PG: Polygalacturonase
PP_333_: Paclobutrazol
qRT-PCR: Quantitative real-time PCR
Swiss-Prot: A manually annotated and reviewed protein sequence database
T_1: The collected flowers on the first day of the late bloom stage with PP_333_ treatment
T_5: The collected flowers on the fifth day of the late bloom stage with PP_333_ treatment
T_9: The collected flowers on the ninth day of the late bloom stage with PP_333_ treatment
ZR: Trans-Zeatin-riboside
