# Supplementary material for: Integrated analysis of mRNA-seq and miRNA-seq in calyx abscission zone of Korla fragrant pear involved in calyx persistence
Source: BMC Plant Biol. 2019 May 9;19:192. doi: 10.1186/s12870-019-1792-0 (PMC6507046; doi:10.1186/s12870-019-1792-0)
Supplement: Supplementary file 1 — Flowers with persistent and deciduous calyx of Korla fragrant pear. (1). Flowers with persistent calyx. (2). Flowers with deciduous calyx. The white arrow points to the separation line of the calyx abscission. (3). The ‘a’ indicate flowers with deciduous calyx, and the calyx have fallen off. The ‘b’ indicate flowers with persistent calyx. (DOCX 2415 kb) [file 12870_2019_1792_MOESM1_ESM.docx]

**Figure S1:** **Flowers with persistent and deciduous calyx of Korla fragrant pear**


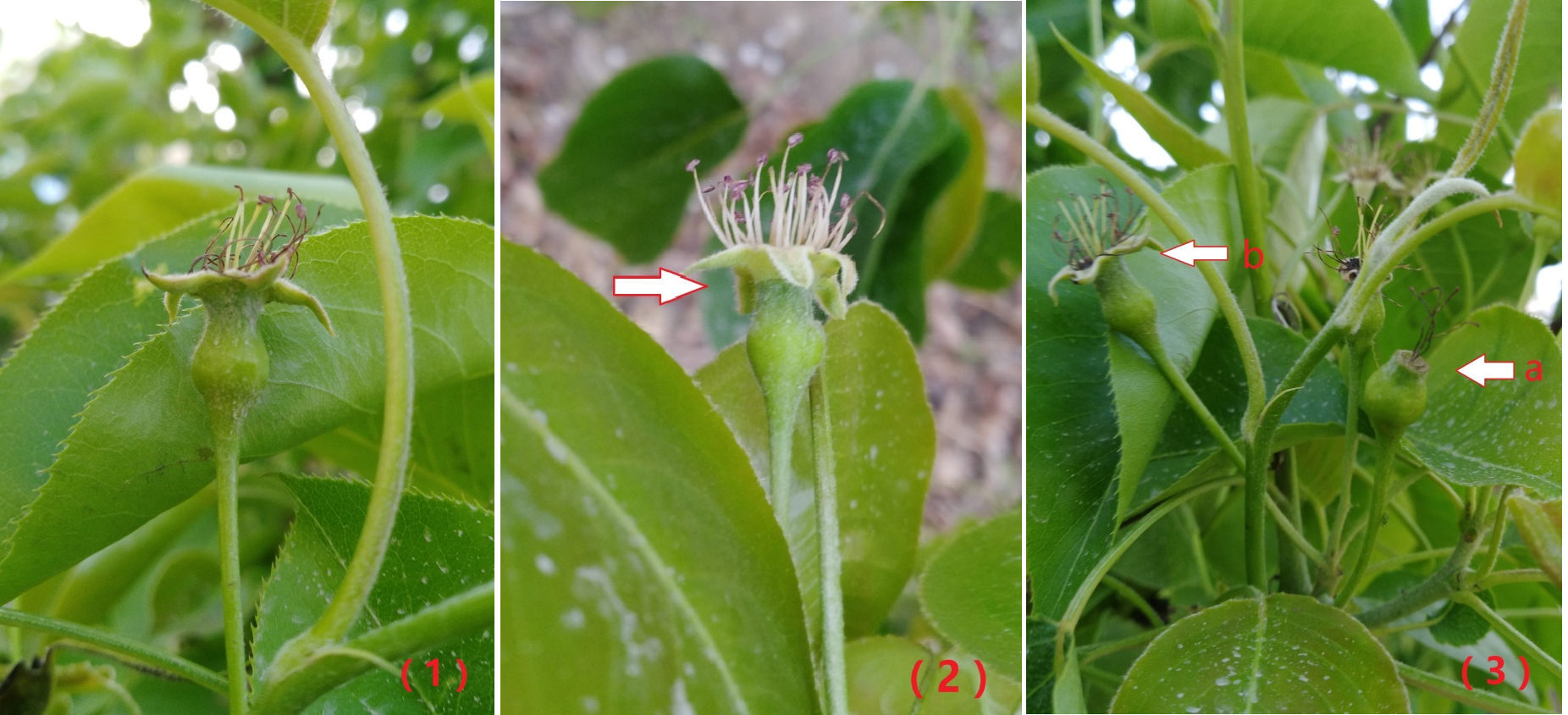


(1). Flowers with persistent calyx. (2). Flowers with deciduous calyx. The white arrow points to the separation line of the calyx abscission. (3). The ‘**a’** indicate flowers with deciduous calyx, and the calyx have fallen off. The ‘**b’** indicate flowers with persistent calyx.
