# Supplementary material for: Integrated analysis of mRNA-seq and miRNA-seq in calyx abscission zone of Korla fragrant pear involved in calyx persistence
Source: BMC Plant Biol. 2019 May 9;19:192. doi: 10.1186/s12870-019-1792-0 (PMC6507046; doi:10.1186/s12870-019-1792-0)
Supplement: Supplementary file 2 — The calyx abscission zone (AZ) tissues samples. a: Sample of Korla fragrant pear flower without petals. b: Sample of calyx abscission zone (AZ) tissues. (DOCX 878 kb) [file 12870_2019_1792_MOESM2_ESM.docx]

**Figure S2: The calyx abscission zone (AZ) tissues samples.**


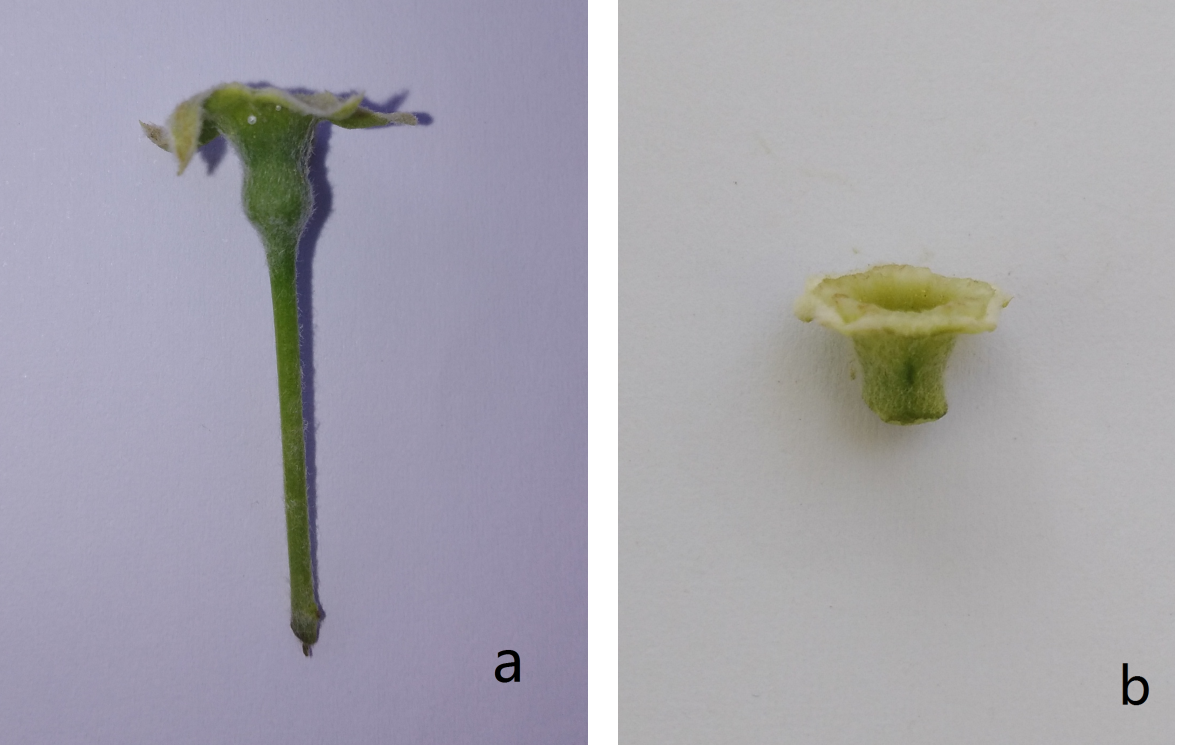


**a**: Sample of Korla fragrant pear flower without petals. **b**: Sample of calyx abscission zone (AZ) tissues.
