# Supplementary material for: Integrated analysis of mRNA-seq and miRNA-seq in calyx abscission zone of Korla fragrant pear involved in calyx persistence
Source: BMC Plant Biol. 2019 May 9;19:192. doi: 10.1186/s12870-019-1792-0 (PMC6507046; doi:10.1186/s12870-019-1792-0)
Supplement: Supplementary file 3 — The qRT-PCR primers. (DOCX 22 kb) [file 12870_2019_1792_MOESM3_ESM.docx]

**Table S1. The qRT-PCR primers**

| Genes | Sequence name | primer sequence（5′–3′） |
| --- | --- | --- |
| psi-miR394a | Forward primer | CGCGTTGGCATTCTGTCCACCTCC |
| psi-miR858b  psi-miR397a  psi-miR396a-5p  psi-miR156j  psi-miR160a-3p  psi-miR167d  psi-miR167a-5p | Forward primer  Forward primer  Forward primer  Forward primer  Forward primer  Forward primer  Forward primer  Reverse primer | ACGCGTTCGTTGTCTGTTCGACCTTG  ACGCGTCATTGAGTGCAGCGTTGATG  CGCGCGTTCCACAGCTTTCTTGAACTG  GCGCGTGACAGAAGAGAGAGAGCAC  GGCGTATGAGGAGCCATGCATA  TGAAGCTGCCAGCATGATCTGG  CGTGAAGCTGCCAGCATGATCTA  ATCCAGTGCAGGGTCCGAGG |
| HAB1(Cluster-7606.44181) | Forward primer  Reverse primer | CCAAGAGGCACGTATGACAGCAG  TCAGGAGCCATAGGTTGGAGAAGG |
| Beta-galactosidase (Cluster-9706.74090) | Forward primer  Reverse primer | GCGGCAAGCCTTCTCAGAGATG  CCGAGAGCTTCTTCTTGTCGTGTC |
| HERK1 (Cluster-9706-30698) | Forward primer  Reverse primer | GAACAACTCAGCAGGTAGCCTCAG  CTTGGTCTGCCAGTCTCTTCCTTC |
| LAC7 (Cluster-9706.1522) | Forward primer  Reverse primer | ATCGGCGTCAACTTGGAGATGTG  CGTTGTTCATGCTTGCGGATAGTC |
| ZAT12 (Cluster-9706.14884) | Forward primer  Reverse primer | GCCACAGAGCGAGTCACAAGAAG  CTGTCCGACCGTGAACTCAAGC |
| SPL13A (Cluster-9706.35292)  ARF6 (Cluster-9706.66959)  ARF18 (Cluster-9706-105236)  ARF25 (Cluster-9706.68003)  CYP707A1(Cluster-9706.109952) | Forward primer  Reverse primer  Forward primer  Reverse primer  Forward primer  Reverse primer  Forward primer  Reverse primer  Forward primer  Reverse primer | CGCAGACGCAGCAGACATCG  GCATCACCACTTCCATTGGAGACC  AGAAGGTGGTACCGATCCTCAAGG  AGAACCACAATCGCCACCAACTC  TTCCGTTCGCATCACCACTACAAG  GGCAGTGGACCTGAATACGCATC  TCCTTCCAGACCGCTACCTACATC  TGAGGCTGAGACTGGCGATGG  AGGATGCAGTTAGAGGAGGAGGAC  GTCAAGACGCTGGCTGTGGTG |
| U6 | Forward primer | CTCGCTTCGGCAGCACA |
|  | Reverse primer | AACGCTTCACGAATTTGCGT |
| Actin | Forward primer | CCATCCAGGCTGTTCTCTC |
|  | Reverse primer | GCAAGGTCCAGACGAAGG |
