# Supplementary material for: Integrated analysis of mRNA-seq and miRNA-seq in calyx abscission zone of Korla fragrant pear involved in calyx persistence
Source: BMC Plant Biol. 2019 May 9;19:192. doi: 10.1186/s12870-019-1792-0 (PMC6507046; doi:10.1186/s12870-019-1792-0)

C_1vsT_1

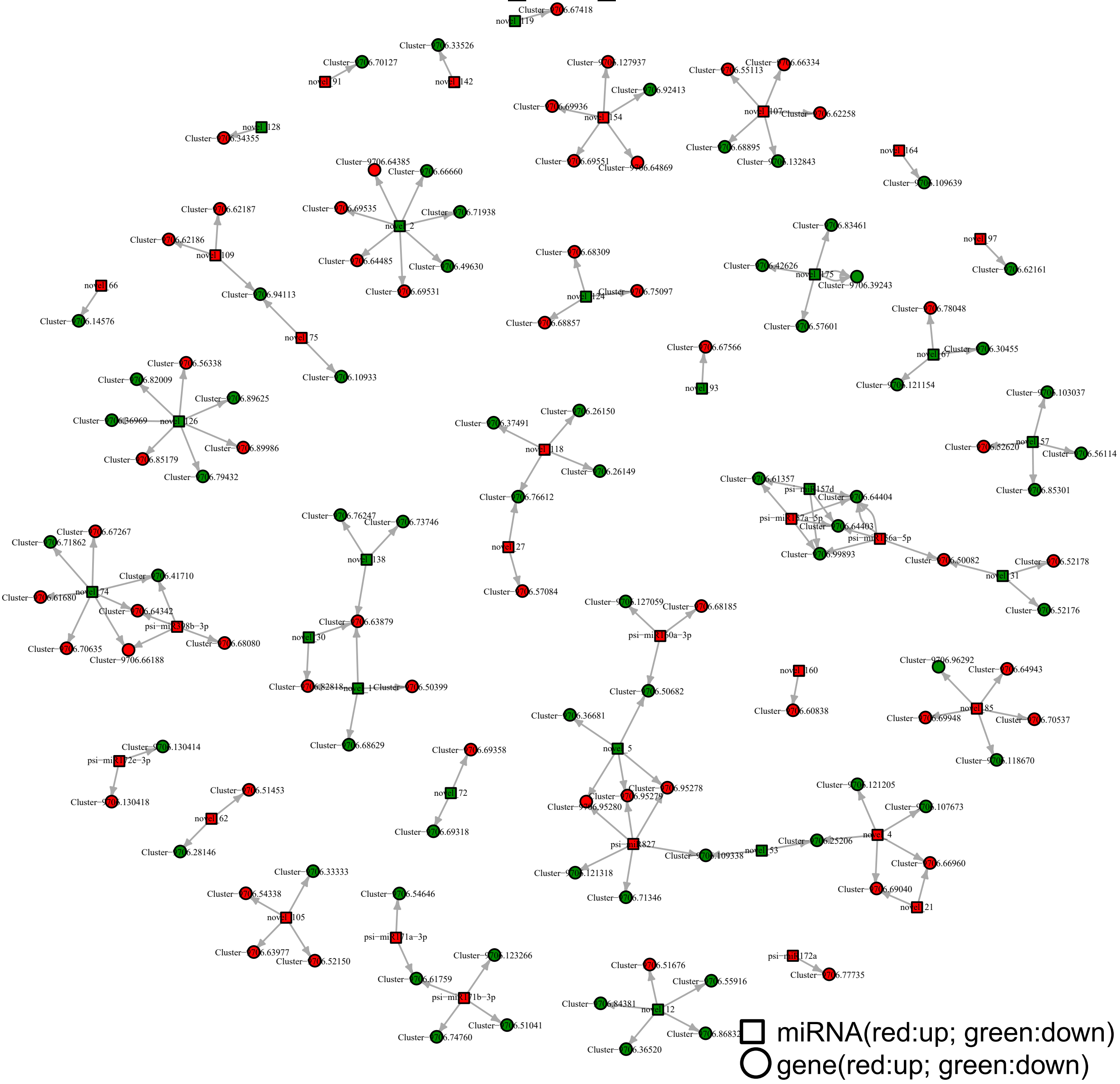

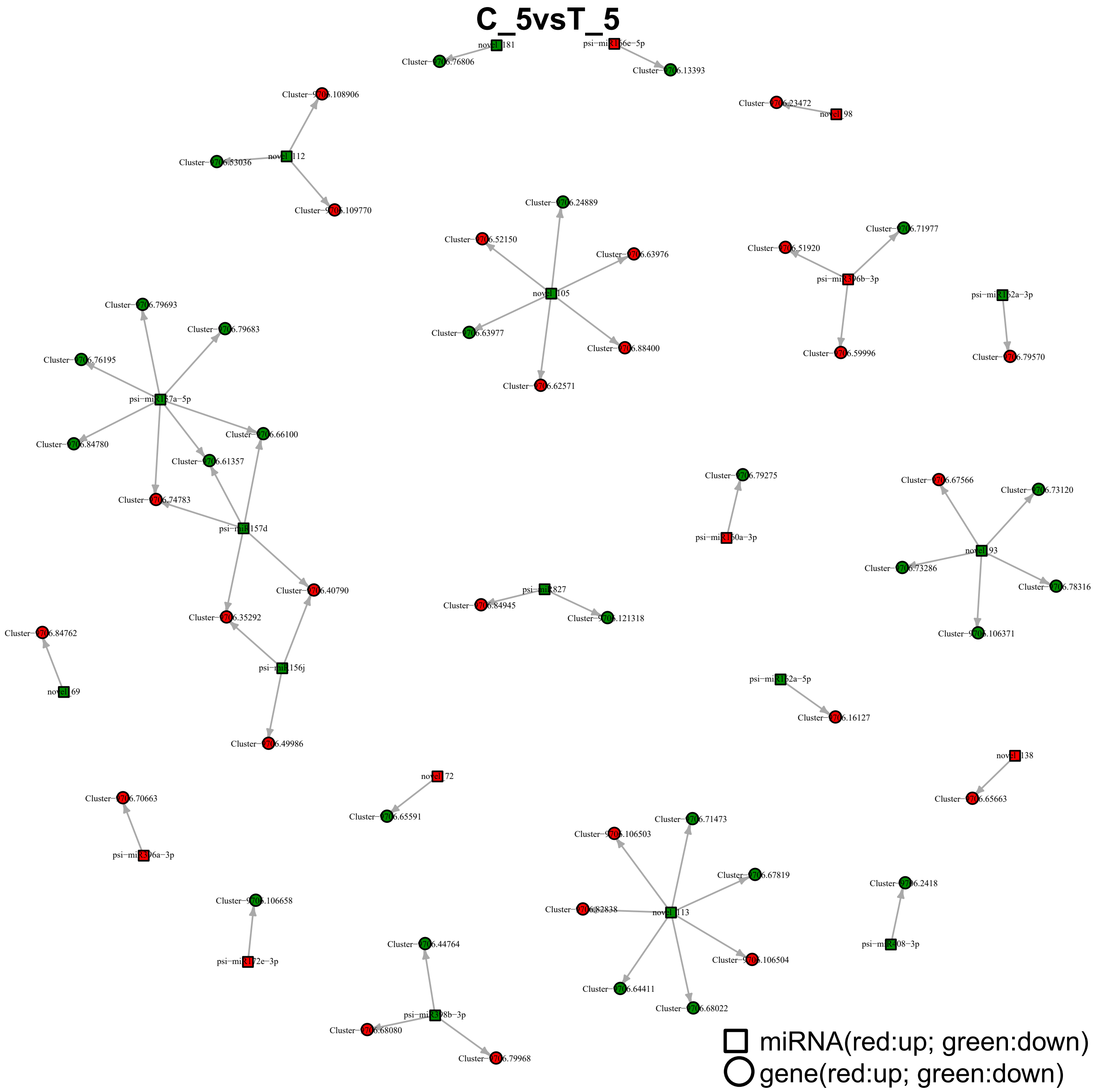
C_5vsT_5
novel_181
Cluster-9706.76806
psi-miR156e-5p
Cluster-9706.13393
Cluster-9706.108906
Cluster-9706.23472
novel_198
novel_112
Cluster-9706.53036
Cluster-9706.109770
Cluster-9706.24889
Cluster-9706.52150
Cluster-9706.63976
novel_105
Cluster-9706.63977
Cluster-9706.88400
Cluster-9706.62571
Cluster-9706.71977
Cluster-9706.51920
psi-miR396b-3p
Cluster-9706.59996
psi-miR162a-3p
Cluster-9706.79570
Cluster-9706.79693
Cluster-9706.79683
Cluster-9706.76195
psi-miR157a-5p
Cluster-9706.84780
Cluster-9706.66100
Cluster-9706.61357
Cluster-9706.74783
psi-miR157d
Cluster-9706.40790
Cluster-9706.35292
psi-miR156j
Cluster-9706.49986
Cluster-9706.84762
novel_169
Cluster-9706.79275
psi-miR160a-3p
Cluster-9706.67566
Cluster-9706.73120
novel_193
Cluster-9706.73286
Cluster-9706.78316
Cluster-9706.106371
psi-miR827
Cluster-9706.84945
Cluster-9706.121318
psi-miR162a-5p
Cluster-9706.16127
novel_138
Cluster-9706.65663
Cluster-9706.70663
psi-miR396a-3p
novel_172
Cluster-9706.65591
Cluster-9706.71473
Cluster-9706.106503
Cluster-9706.67819
Cluster-9706.82838
novel_113
Cluster-9706.106504
Cluster-9706.64411
Cluster-9706.68022
Cluster-9706.2418
psi-miR408-3p
Cluster-9706.106658
psi-miR172e-3p
Cluster-9706.44764
psi-miR398b-3p
Cluster-9706.68080
Cluster-9706.79968
miRNA(red:up; green:down)
gene(red:up; green:down)

C_9vsT_9

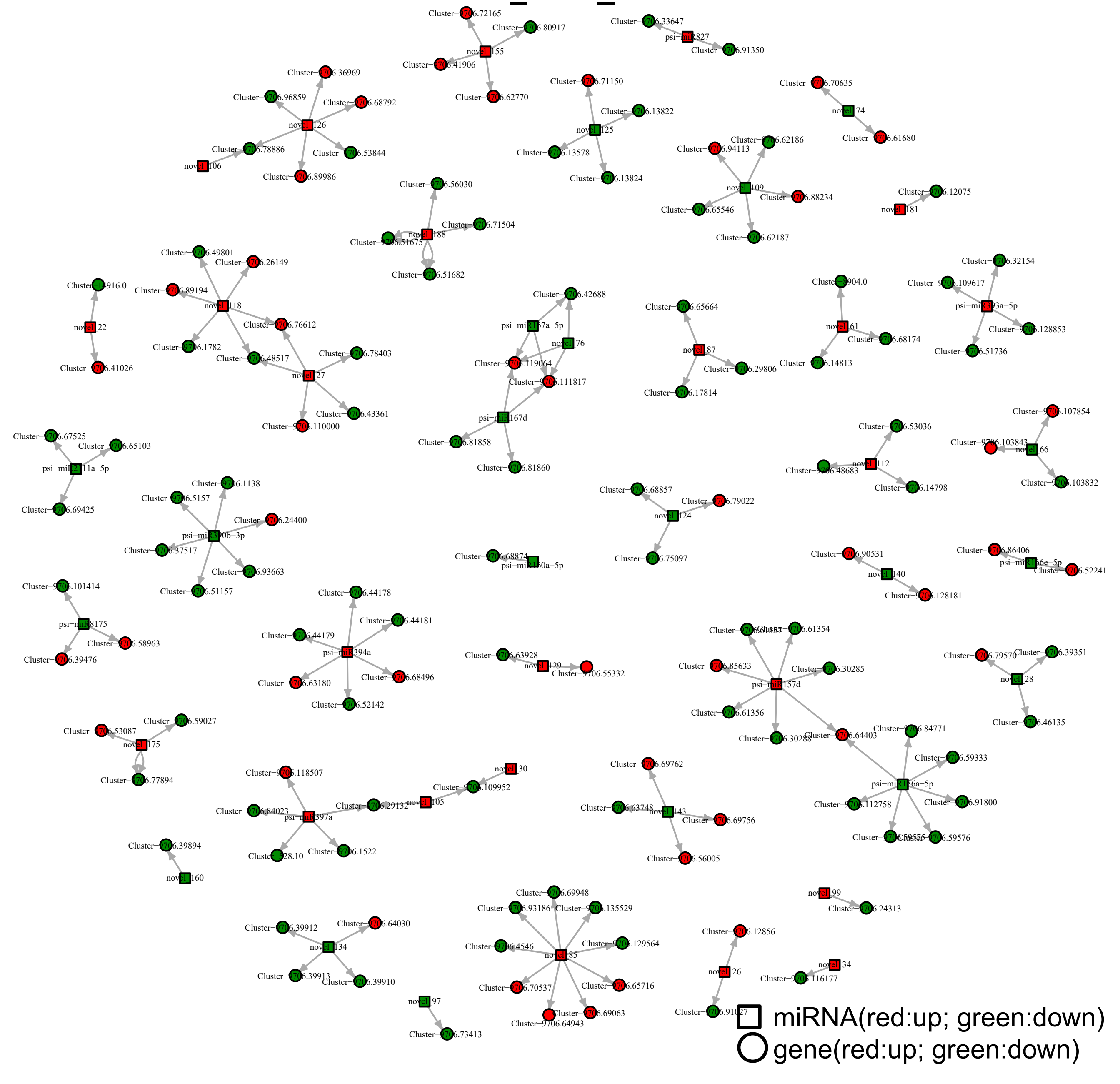

C_1vsC_5

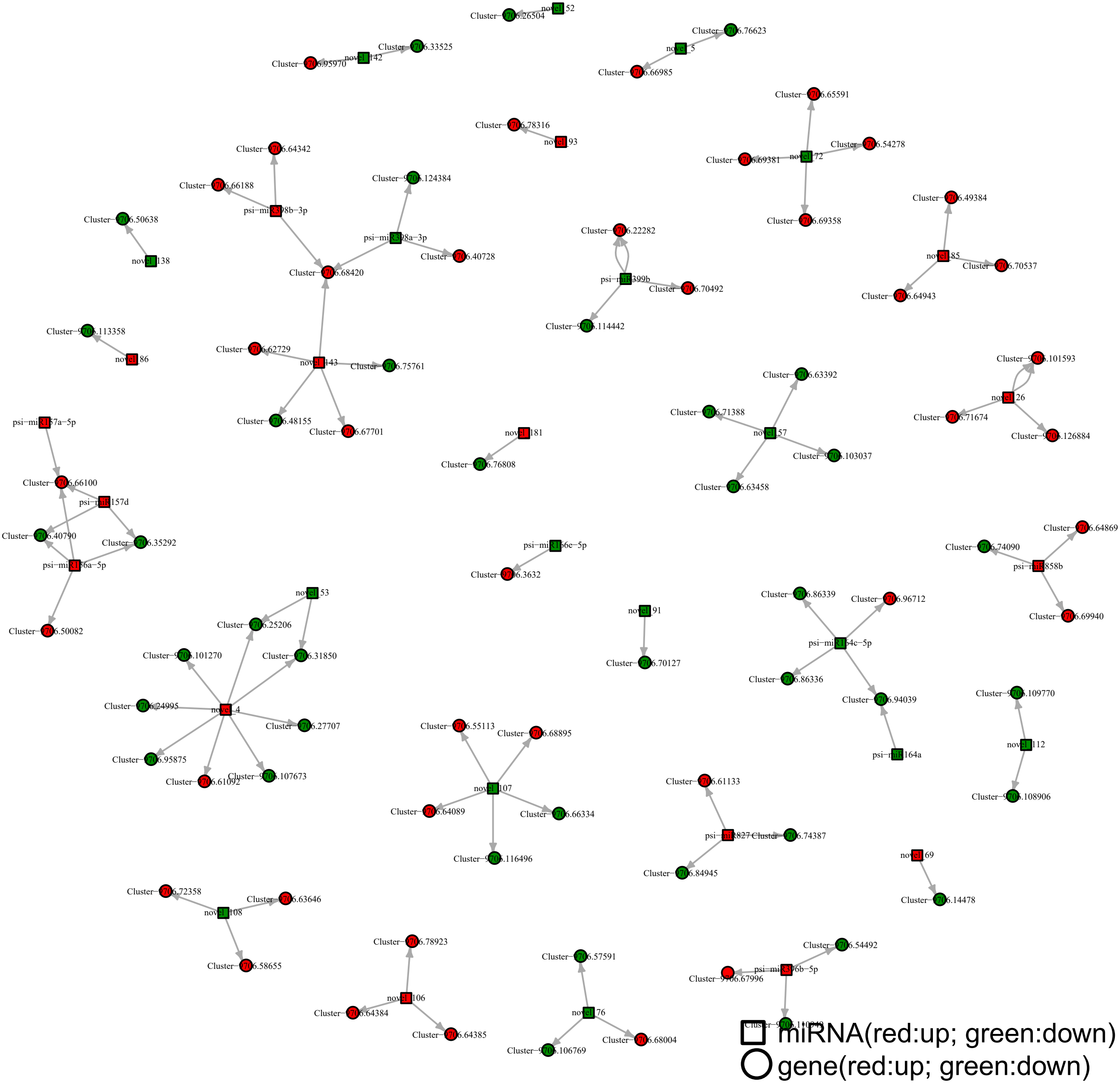

C_1vsC_9

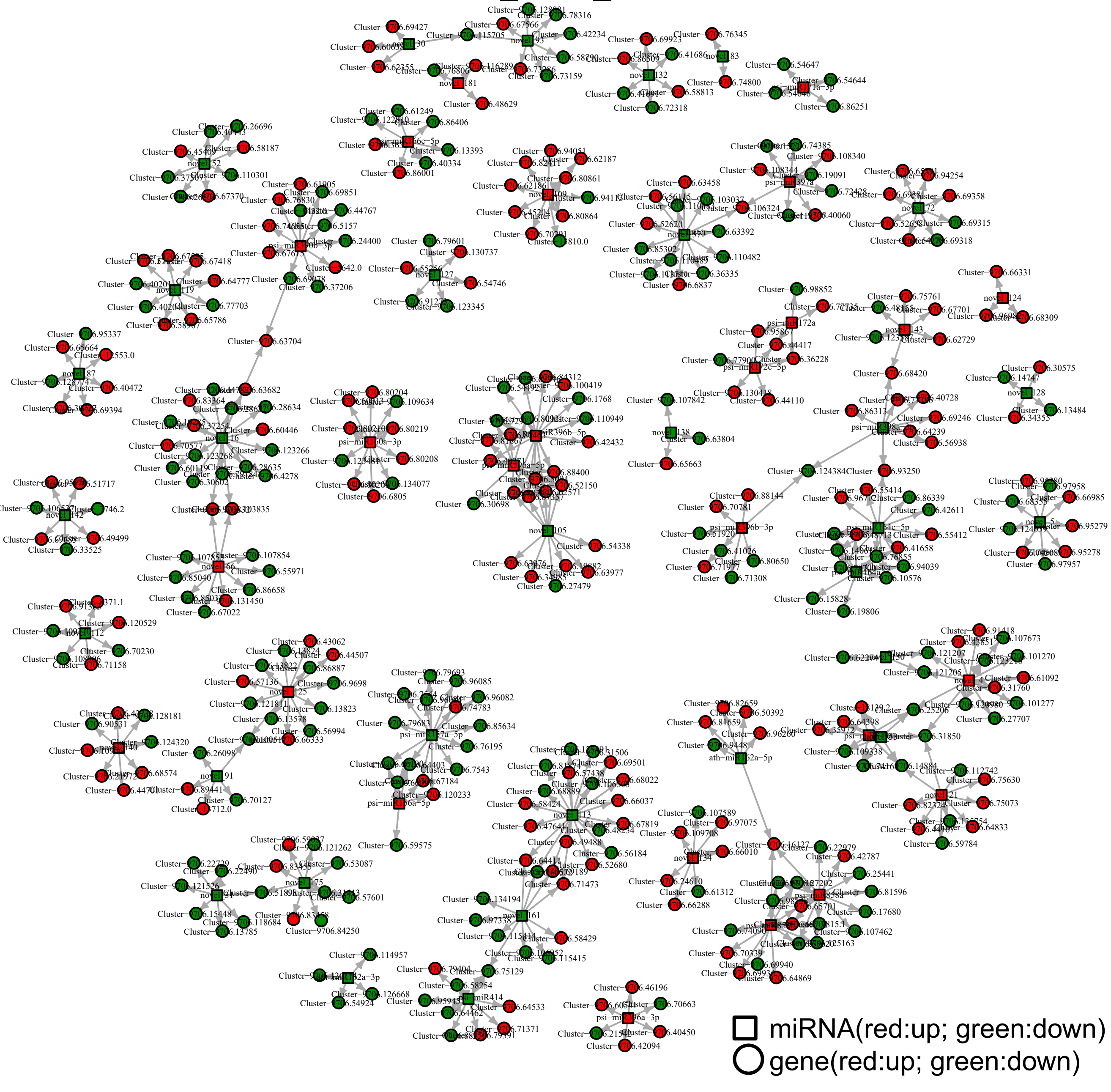

C_5vsC_9

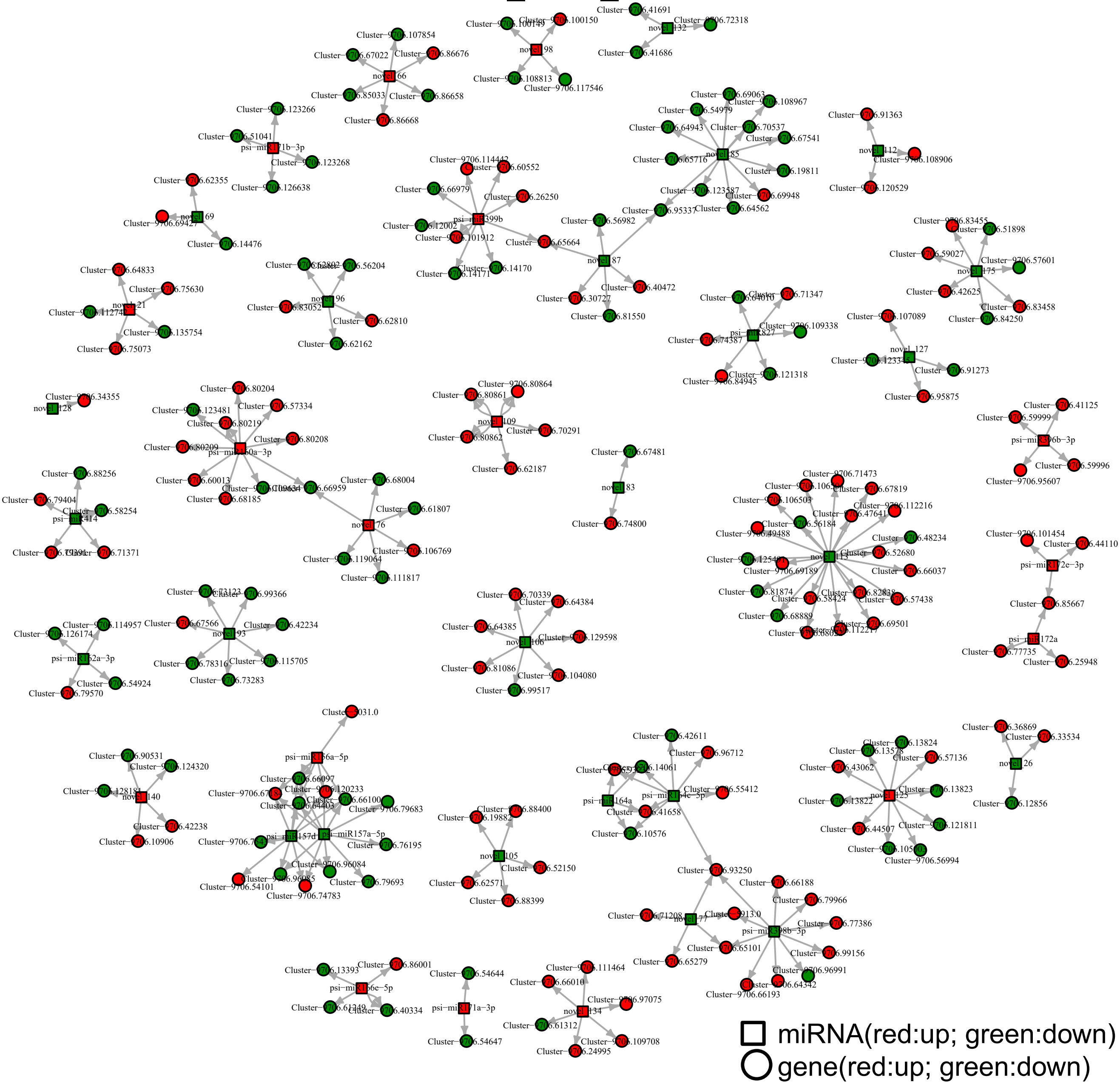

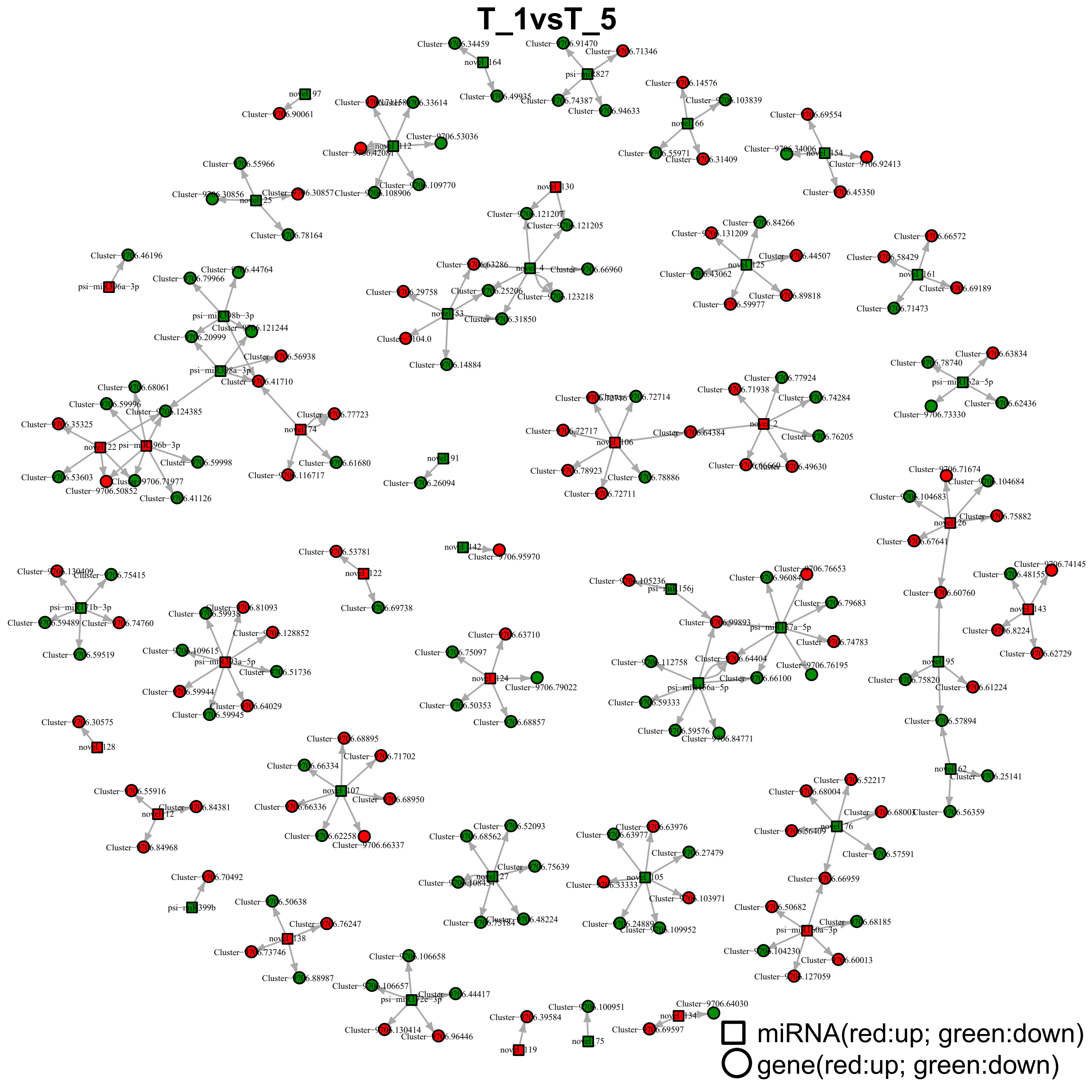
T_1vsT_5
miRNA(red:up; green:down)
gene(red:up; green:down)

T_1vsT_9

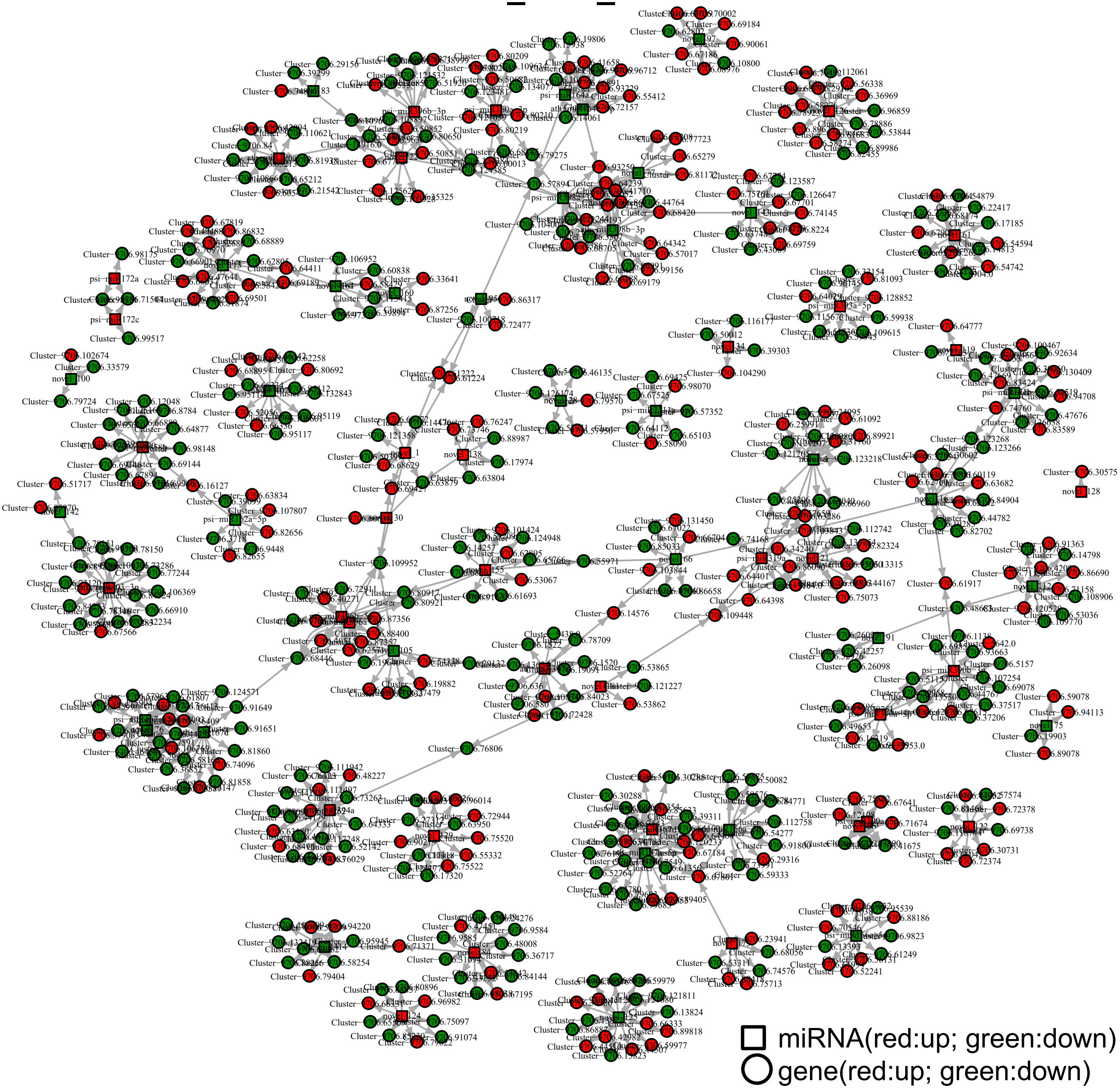

T_5vsT_9

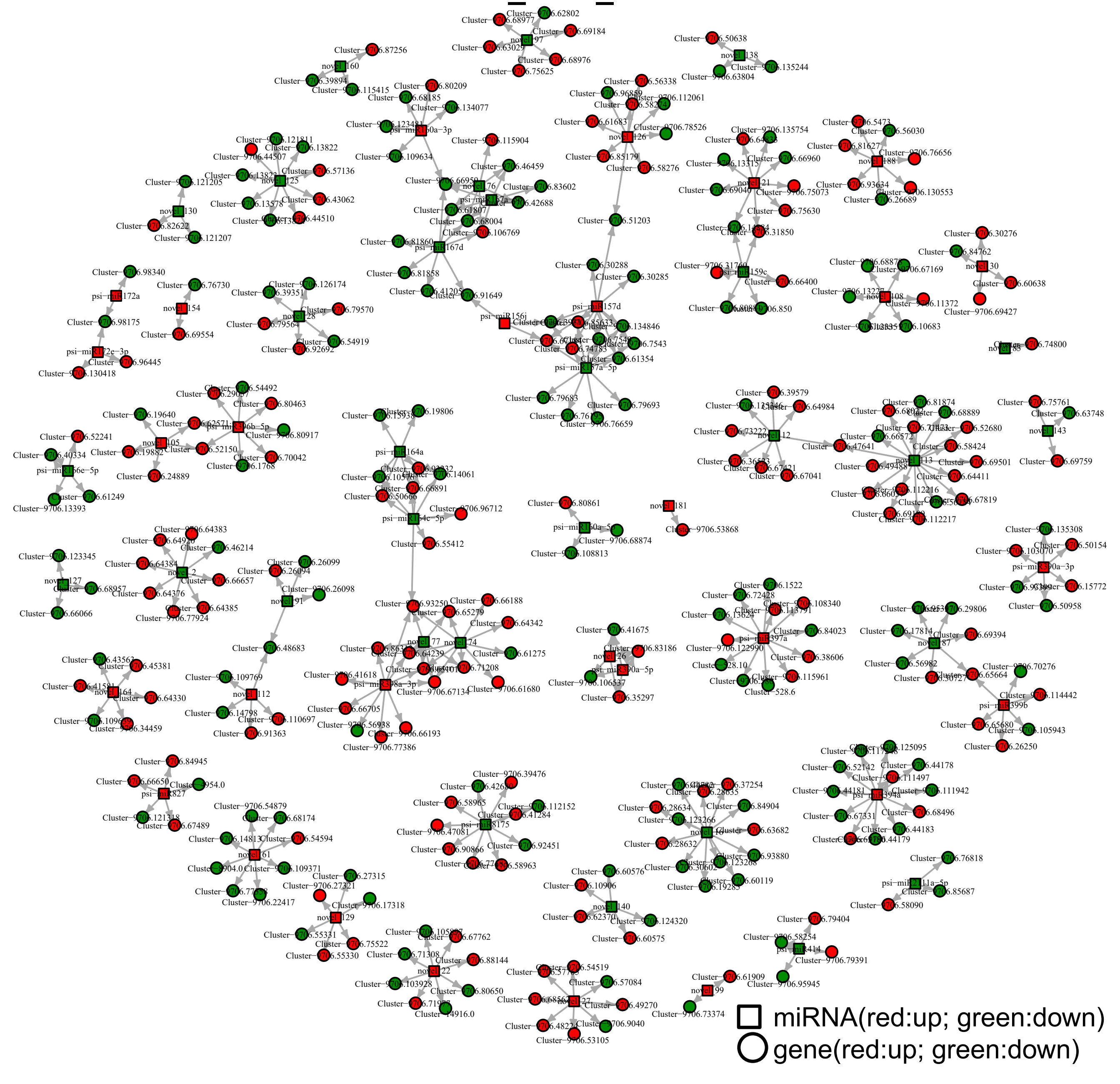

Supplement: Supplementary file 8 — miRNA-mRNA pairs among the six treatment groups. (PDF 4832 kb) [file 12870_2019_1792_MOESM8_ESM.pdf]
